# The Lewis superacid Al[N(C_6_F_5_)_2_]_3_ and its higher homolog Ga[N(C_6_F_5_)_2_]_3_ – structural features, theoretical investigation and reactions of a metal amide with higher fluoride ion affinity than SbF_5_†
†Electronic supplementary information (ESI) available. CCDC 1557072–1557076. For ESI and crystallographic data in CIF or other electronic format see DOI: 10.1039/c7sc03988c


**DOI:** 10.1039/c7sc03988c

**Published:** 2017-10-23

**Authors:** J. F. Kögel, D. A. Sorokin, A. Khvorost, M. Scott, K. Harms, D. Himmel, I. Krossing, J. Sundermeyer

**Affiliations:** a Fachbereich Chemie der Philipps-Universität , Hans-Meerwein Str. , 35043 Marburg , Germany . Email: jsu@staff.uni-marburg.de; b Institut für Anorganische und Analytische Chemie , Freiburger Materialforschungszentrum (FMF) , Freiburg Institute for Advanced Studies (FRIAS) , Section Soft Matter Science , Universität Freiburg , Albertstr. 21 , 79104 Freiburg , Germany

## Abstract

Herein we present the synthesis of the two Lewis acids Al[N(C_6_F_5_)_2_]_3_ (ALTA) and Ga[N(C_6_F_5_)_2_]_3_ (GATA) *via* salt elimination reactions. The metal complexes were characterized by NMR-spectroscopic methods and X-ray diffraction analysis revealing the stabilization of the highly Lewis acidic metal centers by secondary metal–fluorine contacts. The Lewis acidic properties of Al[N(C_6_F_5_)_2_]_3_ and Ga[N(C_6_F_5_)_2_]_3_ are demonstrated by reactions with Lewis bases resulting in the formation of metallates accompanied by crucial structural changes. The two metallates [Cs(Tol)_3_]^+^[FAl(N(C_6_F_5_)_2_)_3_]^–^ and [AsPh_4_]^+^[ClGa(N(C_6_F_5_)_2_)_3_]^–^ contain interesting weakly coordinating anions. The reaction of Al[N(C_6_F_5_)_2_]_3_ with trityl fluoride yielded [CPh_3_]^+^[FAl(N(C_6_F_5_)_2_)_3_]^–^ which could find application in the activation of metallocene polymerization catalysts. The qualitative Lewis acidity of Al[N(C_6_F_5_)_2_]_3_ and Ga[N(C_6_F_5_)_2_]_3_ was investigated by means of competition experiments for chloride ions in solution. DFT calculations yielded fluoride ion affinities in the gas phase (FIA) of 555 kJ mol^–1^ for Al[N(C_6_F_5_)_2_]_3_ and 472 kJ mol^–1^ for Ga[N(C_6_F_5_)_2_]_3_. Thus, Al[N(C_6_F_5_)_2_]_3_ can be considered a Lewis superacid with a fluoride affinity higher than SbF_5_ (493 kJ mol^–1^) whereas the FIA of the corresponding gallium complex is slightly below the threshold to Lewis superacidity.

## Abstract 2

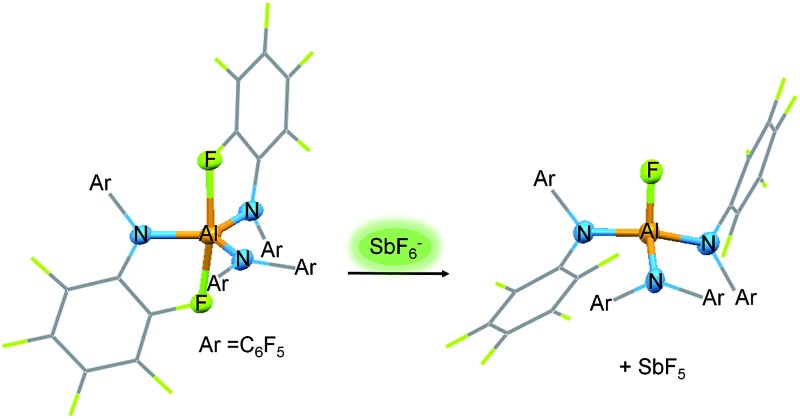
Lewis superacid with higher fluoride ion affinity than SbF_5_.

## Introduction

Lewis acidic compounds play an important role in synthetic chemistry and have been successfully applied to Diels–Alder reactions,^1^ rearrangements,^2^ conjugate additions^3^ or Friedel–Crafts reactions^4^ to name only a few examples. Thus, Lewis acid catalysis has been the subject of various review articles^5^ and the scientific activity in the field of Lewis acids was additionally kindled by the development of frustrated Lewis pair chemistry by Stephan in 2006.^6^

**Chart 1:**
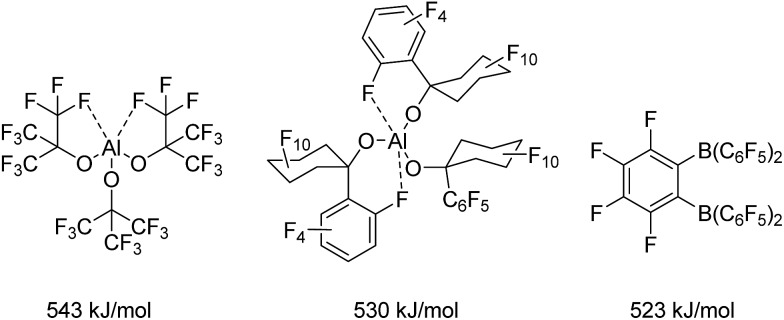
Examples for Lewis superacids and their calculated FIAs.

The importance of Lewis acids as valuable synthetic tools has evoked a fundamental interest in the phenomenon of Lewis acidity and its underlying principles. In this context, Haartz and McDaniel in 1973 introduced the fluoride ion affinity in the gas phase (FIA) as the benchmark for the quantification of Lewis acidity.^7^ Bartlett *et al.* took up on this and extended the scale.^8^,^9^ Christe and Dixon were the first to introduce a reliable isodesmic calculation recipe for the FIA.^10^ However, the first FIA value (without naming it as such) was presented for BF_3_ already in 1955.^11^ By definition, SbF_5_ as the strongest conventional molecular Lewis acid with a calculated FIA of 493 kJ mol^–1^ 12 marks the threshold to Lewis superacidity. Krossing *et al.* reported on the preparation of the fluorobenzene adduct of the homoleptic aluminum complex Al[OC(CF_3_)_3_]_3_ (Chart 1, FIA: 505 kJ mol^–1^ in case of the PhF adduct^8^ and 543 kJ mol^–1^ for the corresponding adduct free form^9^) and highlighted important requirements for the design of Lewis superacids: The generation of an extremely electron-poor metal center can be achieved by ligands with weak donor properties that usually contain strongly electron withdrawing substituents such as perfluorinated alkyl groups. In case of Al[OC(CF_3_)_3_]_3_, the aluminum center is stabilized by the formation of two hemilabile aluminum–fluorine interactions masking the high Lewis acidity of the metal center. These metal–fluorine contacts break up in the presence of a Lewis base. The incorporation of additional O- or N-donor atoms in the ligand backbone instead of the carbon-bonded fluorine atoms would allow the formation of stable chelates, which drastically reduce the Lewis acidic properties of the metal complex. Furthermore, sufficient bulkiness of the ligand moieties should prevent oligomerization which would have a reducing effect on the Lewis acidity and complicate the theoretical determination of the FIA. Such decrease in the Lewis acidity due to aggregation is observed for aluminum triiodide and aluminum tribromide that reach the demanded FIA for Lewis superacidity in their monomeric forms in the gas phase (AlI_3_: 535 kJ mol^–1^ AlBr_3_: 510 kJ mol^–1^),^9^ but show dramatically lower values in the solid state (AlI_3_: 429 kJ mol^–1^ AlBr_3_: 408 kJ mol^–1^) because of their high monomerisation enthalpies of 106 kJ mol^–1^ and 102 kJ mol^–1^, respectively.^13^ Eventually, the ligand regime has to provide inertness towards intramolecular or intermolecular degradation processes like the abstraction of fluorine atoms from the ligand backbone. Beside Al[OC(CF_3_)_3_]_3_, the related Al[O(C_6_F_10_(C_6_F_5_))]_3_ (530 kJ mol^–1^)^14^ also meets the criterion for Lewis superacidity. Lately Wiesner *et al.* revealed the enormous Lewis acidity of Al(OTeF_5_)_3_ which could be isolated as an acetonitrile adduct.^15^

Among perfluorinated aluminum aryl Lewis acids, Al(C_6_F_5_)_3_ is the most prominent exhibiting an FIA of 530 kJ mol^–1^.^8^,^16^ It has been tested in metallocene^17^ and alkyne activation reactions^18^ as well as a component of weakly coordinating anions (WCAs).^19^ Only recently Chen and Chen reported a [Si–H···Al] interaction in a crystal structure of [Et_3_Si-H-Al(C_6_F_5_)_3_]^20^ in analogy to works by Piers and Tuononen^21^ and Stephan^22^ who demonstrated [Si–H···B] interactions. However, despite its considerably higher Lewis acidity, the explosive Al(C_6_F_5_)_3_ has received less attention than the corresponding boron compound.^13^ Whereas common boranes like the widely used B(C_6_F_5_)_3_ (452 kJ mol^–1^) show FIAs below the threshold to Lewis superacidity, only the chelating 1,2-[(C_6_F_5_)_2_B]_2_C_6_F_4_ (523 kJ mol^–1^) exhibits an FIA higher than that of SbF_5_.^9^,^23^ Very recently the group of Mitzel published tris(perfluorotolyl)boran which turned out to be more Lewis acidic than its parent compound B(C_6_F_5_)_3_.^24^,^25^

The design of highly Lewis acidic metal complexes has also been the subject of theoretical works.^26^ Frenking *et al.* reported on the enhancement of the Lewis acidity of B, Al and Ga compounds with adamantyl substituents by pyramidalization of the coordination geometry.^27^

The research in the field of strong Lewis acids goes hand in hand with the investigation of the corresponding weakly coordinating anions derived from the reaction of a Lewis acid with a Lewis basic anion. The metal center is shielded by the hydrophobic and sterically demanding perfluorinated ligand regime granting delocalization of the negative charge. Thus, WCAs allow the stabilization of highly reactive cationic species^28^ like the carbocations [CCl_3_]^+^ and [CBr_3_]^+^,^29^ the tritylium cation,^30^ a radical cation of benzidine,^31^ a stable [AsBr_4_]^+^ cation^32^ or a [Ag_2_Se_12_]^2+^ cage.^33^ In this context, especially Ag^+^[Al(OC(CF_3_)_3_)_4_]^–^ has emerged as a versatile reagent for the abstraction of chloride ions from neutral precursors to generate reactive cations stabilized by the WCA [Al(OC(CF_3_)_3_)_4_]^–^.^34^ Such reactions yielded stabilized amido-substituted germanium(ii) and tin(ii) monocations,^35^ homoleptic ethylene complexes of the coinage metals,^36^ the *t*Bu_3_Si^+^ source [*t*Bu_3_Si–Ga–Si*t*Bu_3_]^+^,^37^ gallium(i) arene complexes^38^ or univalent gallium and indium phosphane complexes.^39^

Aluminum and gallium are proper metals for the generation of strong Lewis acids because of the small size and the high charge of their M^3+^ cations. The principle for the preparation of Lewis acidic aluminum or gallium compounds is to find a negatively charged ligand with weak donor character leaving a high positive partial charge on the metal center. This can be achieved by delocalizing the ligand's negative charge over perfluorinated electron withdrawing groups. As described above, it has been demonstrated that perfluorinated alkoxo ligands are able to form Lewis superacidic aluminum complexes. This article is concerned with the question: can certain perfluorinated metal amides be Lewis superacids and display a higher fluoride affinity than SbF_5_? Representative amido ligands of intrinsically weak donor capability, [N(C_6_F_5_)(C(CF_3_)_3_)]^–^ 40 and [N(C_6_F_5_)(SO_2_R^F^)]^–^ 41 were introduced by us only recently. In this context, we also turned our attention to bis(pentafluorophenyl)amide [N(C_6_F_5_)_2_]^–^ as a promising ligand for the preparation of strong Lewis acids.^42^ HN(C_6_F_5_)_2_ can be easily prepared in large scale^43^ and its two strongly electron withdrawing pentafluorophenyl substituents should provide complexes with good solubility in nonpolar solvents and sufficient sterical shielding of the metal center. Furthermore, the NH-acid is known for its ability to form hemilabile metal–fluorine contacts *via* its *ortho*-fluorine atoms stabilizing the metal center and leading to interesting coordination modes. It has already been incorporated into complexes of lithium,^44^ the f-block metals neodymium,^45^ cerium, lanthanum^46^ and uranium^47^ and the d-block metals titanium, zirconium, vanadium, iron, cobalt^48^ and tungsten.^49^

## Results and discussion

### Preparation

**Scheme 1:**
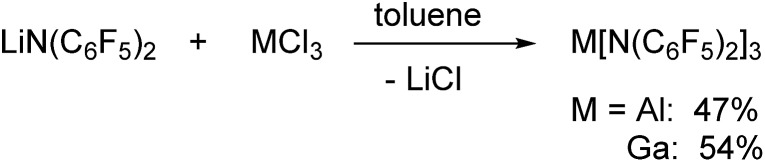
Preparation of Al[N(C_6_F_5_)_2_]_3_ and Ga[N(C_6_F_5_)_2_]_3_*via* salt elimination reactions.

Al[N(C_6_F_5_)_2_]_3_ and Ga[N(C_6_F_5_)_2_]_3_ were both prepared *via* reactions of LiN(C_6_F_5_)_2_ with the corresponding metal trichlorides in toluene at 90 °C leading to the precipitation of lithium chloride (Scheme 1). Al[N(C_6_F_5_)_2_]_3_ was first isolated from an alkane elimination reaction between trimethylaluminum and HN(C_6_F_5_)_2_ in toluene at 105 °C, but this route only yielded traces of the desired product. The ^19^F NMR spectra of Al[N(C_6_F_5_)_2_]_3_ (*δ* = –153.1, –158.6 and –161.1 ppm) and Ga[N(C_6_F_5_)_2_]_3_ (*δ* = –151.8, –158.1 and –161.4 ppm) in [D_6_]benzene reveal three signals with similar chemical shifts in a 2 : 1 : 2 ratio for the three aromatic fluorine atoms. As expected, especially the aluminum compound turned out to be extremely moisture-sensitive.

### Structural features of the free Lewis acids

**Chart 2:**
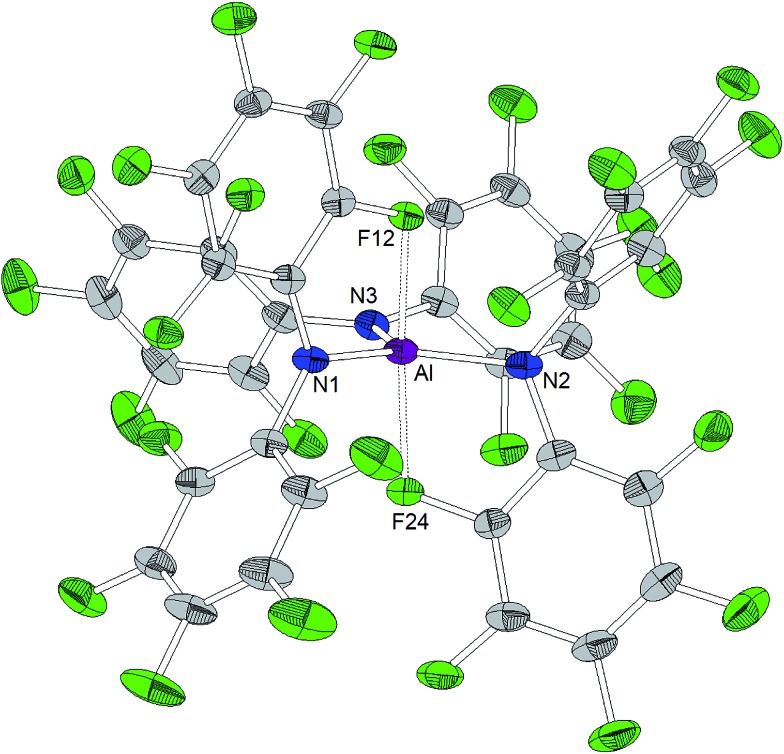
Molecular structure of Al[N(C_6_F_5_)_2_]_3_ (ellipsoids with 30% probability). Selected bond lengths/Å and angles/°: Al–N1 1.843(2), Al–N2 1.840(2), Al–N3 1.805(2), Al···F12 2.084(1), Al···F24 2.060(1), N1–Al–N2 123.7(1), N2–Al–N3 116.2(1), N3–Al–N1 120.09(9), F24–Al–F12 164.93(6).

A trigonal planar AlN_3_ coordination geometry is found for Al[N(C_6_F_5_)_2_]_3_ with Al–N distances of 1.843(2) Å, 1.840(2) Å and 1.805(2) Å (Chart 2). The metal center is further stabilized by two axial aluminum–fluorine contacts with Al···F distances of 2.084(1) and 2.060(1) Å and an F12–Al–F24 angle of 164.93(6)°. The incorporation of the two *ortho*-fluorine atoms in Al···F contacts leads to an elongation of the corresponding C–F bonds (1.3897(2) and 1.3867(1) Å compared to 1.3459(2) and 1.3480(2) Å found for the two other C–F_*ortho*_ bond lengths in the corresponding C_6_F_5_ rings). Similar to the κ-N_3_F_2_ configuration experimentally verified for this aluminum trisamide, a κ-O_3_F_2_ configuration with longer Al···F contacts (2.143 and 2.155 Å) was proposed for the alkoxido superacid Al[OC(CF_3_)_3_]_3_ on the basis of DFT calculations.^8^

**Chart 3:**
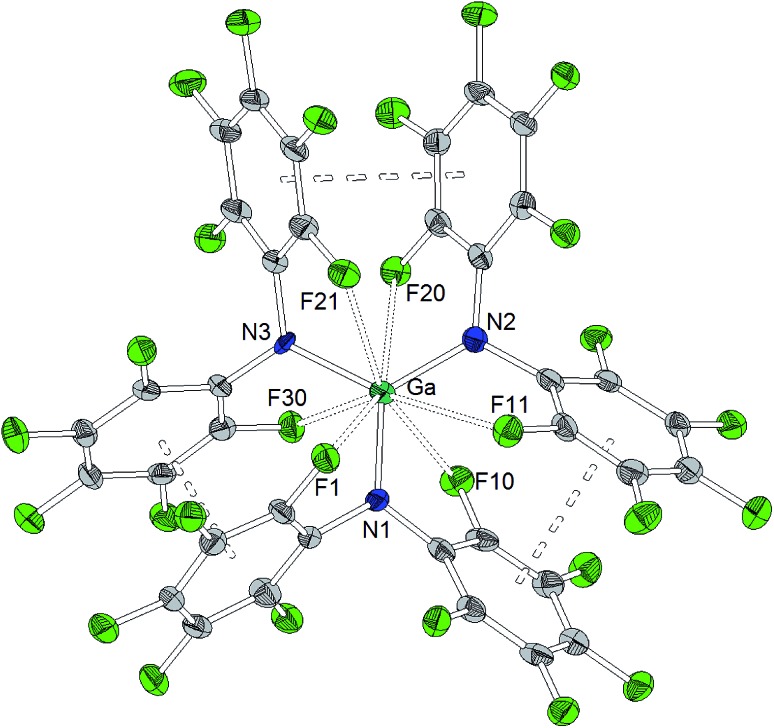
Molecular structure of Ga[N(C_6_F_5_)_2_]_3_ (ellipsoids with 30% probability). Selected bond lengths/Å and angles/°: Ga–N1 1.826(5), Ga–N2 1.798(5), Ga–N3 1.848(5), Ga···F1 3.032(4), Ga···F10 2.981(4), Ga···F11 3.096(4), Ga···F20 2.994(4), Ga···F21 3.102(4), Ga···F30 2.914(4), N1–Ga–N2 122.8(2), N2–Ga–N3 123.0(2), N3–Ga–N1 114.3(2).

As observed for the corresponding aluminum complex, the molecular structure of Ga[N(C_6_F_5_)_2_]_3_ (Chart 3) reveals a trigonal planar GaN_3_ coordination geometry with Ga–N bond lengths of 1.826(5), 1.798(5) and 1.848(5) Å. These values are shorter than the M–F distances found in the molecular structure of Al[N(C_6_F_5_)_2_]_3_. In addition to the three nitrogen donors, the gallium atom is coordinated by six *ortho*-fluorine atoms with gallium–fluorine distances ranging from 2.914(4) to 3.102(4) Å. The complex is further stabilized by π-stacking interactions between the pentafluorophenyl rings of neighboring N(C_6_F_5_)_2_ moieties (distances of neighboring rings' centroids: 3.5192(3), 3.7848(4) and 3.5463(3) Å). Similar coordination modes were observed for the homoleptic lanthanum and cerium complexes of HN(C_6_F_5_)_2_ recently reported by Yin *et al.*^46^ La[N(C_6_F_5_)_2_]_3_ and Ce[N(C_6_F_5_)_2_]_3_ show longer N–M bond lengths (La–N between 2.410(2) and 2.512(2) Å, Ce–N between 2.406(3) and 2.430(2) Å), but shorter M···F contacts (La···F between 2.6695(17) and 2.8942(16) Å, Ce···F between 2.6764(16) and 2.7064(17) Å) compared to Ga[N(C_6_F_5_)_2_]_3_. According to a review article on interactions between metal atoms and organically bound fluorine atoms by Plenio, this kind of coordination mode is one of the two recurrent structure motives for gallium complexes with fluorinated ligands.^50^ The coordination of a gallium center by six fluorine atoms incorporated in the organic ligand backbone was also observed in case of tris(2,4,6-tris(trifluoromethyl)phenyl)gallium exhibiting Ga···F distances between 2.683 and 2.821 Å.^51^

### Reactivity and experimental fluoride ion affinity of Al[N(C_6_F_5_)_2_]_3_

**Scheme 2:**
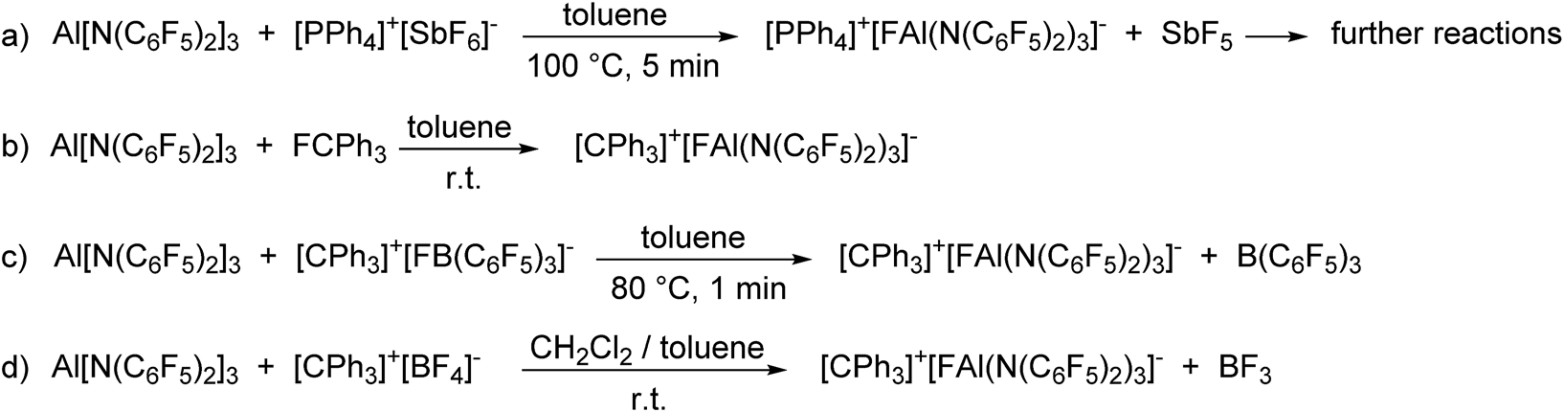
Reactions proving the high Lewis acidity of Al[N(C_6_F_5_)_2_]_3_.

The enormous fluoride ion affinity of Al[N(C_6_F_5_)_2_]_3_ could also be demonstrated experimentally. The reaction of Al[N(C_6_F_5_)_2_]_3_ with [PPh_4_]^+^[SbF_6_]^–^ in toluene at 100 °C resulted in the precipitation of a mixture of [PPh_4_]^+^[FAl(N(C_6_F_5_)_2_)_3_]^–^ and the formation of HN(C_6_F_5_)_2_ (Scheme 2(a)). The ^19^F NMR spectrum of [PPh_4_]^+^[FAl(N(C_6_F_5_)_2_)_3_]^–^ exhibits signals with chemical shifts of –149.6, –166.2, –167.5 and –172.3 ppm in a 12 : 6 : 12 : 1 ratio. The formation of HN(C_6_F_5_)_2_ is plausible as it is known, that SbF_5_ reacts with toluene to give (*p*-Tol)_3_SbF_2_ and three equivalents of HF in moderate yields.^52^ The lewis acid Al[N(C_6_F_5_)_2_]_3_ as well as the corresponding anion [FAl(N(C_6_F_5_)_2_)_3_]^–^ can be protolyzed by HF under formation of HN(C_6_F_5_)_2_. Further proofs for the high fluoride affinity of Al[N(C_6_F_5_)_2_]_3_ could be obtained when dissolving the Lewis acid in hexafluorobenzene or F_2_ClCCF_2_Cl. Both solutions slowly turn dark and the ^19^F NMR spectrum of both reaction mixtures reveal unselective reactions which can be referred to the abstraction of F^–^ from the solvent leading to further reactions of the highly reactive carbocations.

The reaction of Al[N(C_6_F_5_)_2_]_3_ with trityl fluoride in toluene yields [CPh_3_]^+^[FAl(N(C_6_F_5_)_2_)_3_]^–^ which is stable in solution for at least three days (Scheme 2(b)). Attempts to isolate the yellow compound resulted in its decomposition after 24 h at room temperature. In agreement with the theoretically predicted trend of the FIA in the gas phase, [CPh_3_]^+^[FAl(N(C_6_F_5_)_2_)_3_]^–^ is also formed when reacting Al[N(C_6_F_5_)_2_]_3_ with [CPh_3_]^+^[FB(C_6_F_5_)_3_]^–^ in toluene and with [CPh_3_]^+^[BF_4_]^–^ in a mixture of toluene and dichloromethane (Scheme 2(c) and (d)).

**Scheme 3:**
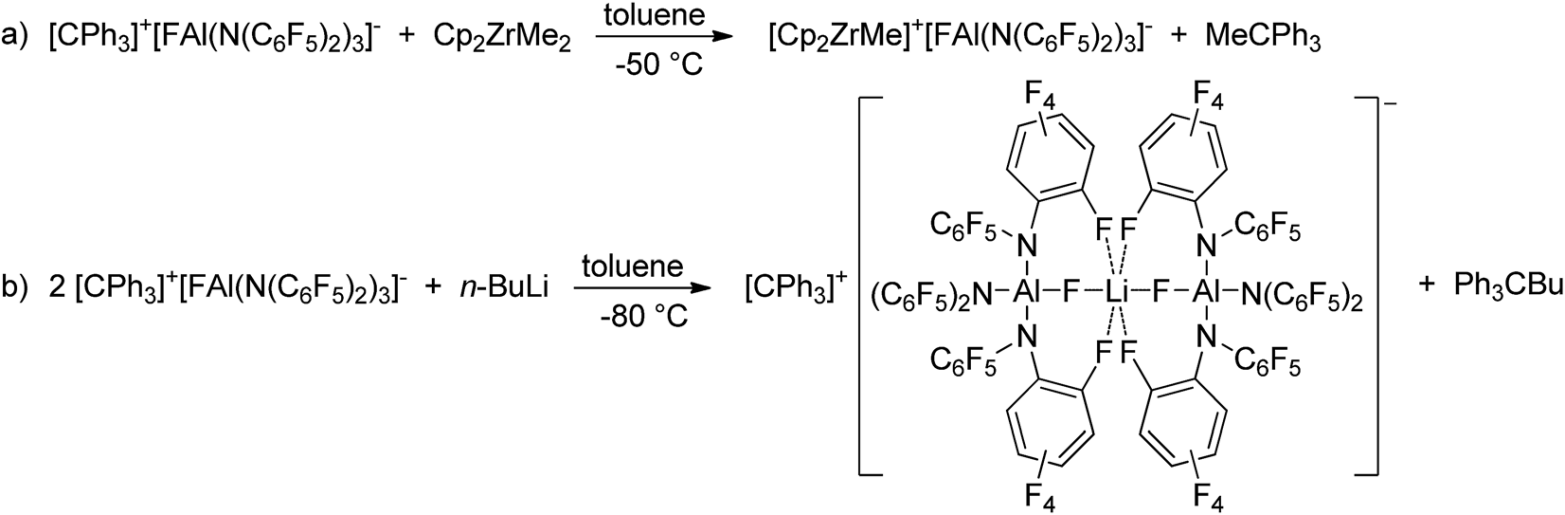
Reactions of [CPh_3_]^+^[FAl(N(C_6_F_5_)_2_)_3_]^–^ with Cp_2_ZrMe_2_ and *n*-butyllithium.

*In situ* generated [CPh_3_]^+^[FAl(N(C_6_F_5_)_2_)_3_]^–^ was able to abstract a methyl group from dimethyl zirconocene to give [Cp_2_ZrMe]^+^[FAl(N(C_6_F_5_)_2_)_3_]^–^ as a white solid (Scheme 3(a)). The ^19^F NMR spectrum shows four signals with chemical shifts of –148.1, –149.4, –160.6 and –163.5 ppm in a 1 : 12 : 6 : 12 ratio. The aluminum bound fluorine atom is shifted to lower field compared to [FAl(N(C_6_F_5_)_2_)_3_]^–^ (*δ* = –170.8 ppm). This can be referred to the coordination of the fluorine atom to the zirconium center which was also observed in a low quality crystal structure of [Cp_2_ZrMe]^+^[FAl(N(C_6_F_5_)_2_)_3_]^–^. The strong Zr···F interaction explains why [Cp_2_ZrMe]^+^[FAl(N(C_6_F_5_)_2_)_3_]^–^ turned out to be inactive in ethene polymerization reactions.

**Chart 4:**
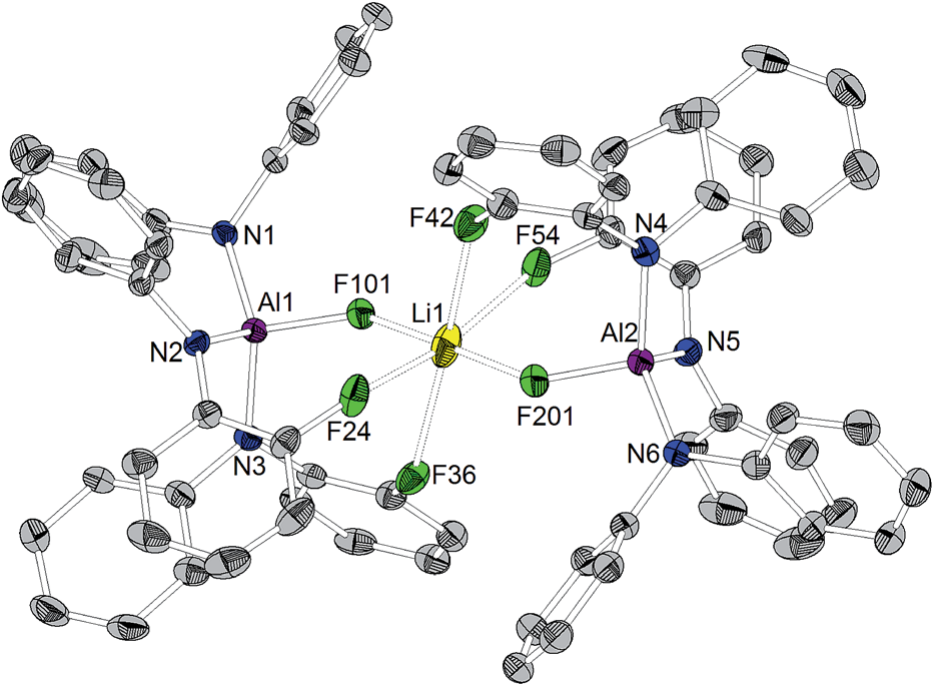
Molecular structure of [CPh_3_]^+^[((C_6_F_5_)_2_N)_3_AlF–Li–FAl(N(C_6_F_5_)_2_)_3_]^–^ (ellipsoids with 30% probability, non-coordinating fluorine atoms, the cation and two toluene molecules omitted for clarity). Selected bond lengths/Å and angles/°: Al1–N1 1.862(3), Al1–N2 1.859(4), Al1–N3 1.857(3), Al1–F101 1.714(3), Al2–N4 1.865(4), Al2–N5 1.859(4), Al2–N6 1.861(4), Al2–F201 1.710(3), Li1···F101 1.793(9), Li1···F201 1.809(9), Li1···F24 2.185(12), Li1···F36 2.392(10), Li1···F42 2.280(10), Li1···F54 2.287(12), N1–Al1–N2 113.51(15), N2–Al1–N3 102.24(16), N1–Al1–N3 123.30(16), N1–Al1–F101 99.83(15), N2–Al1–F101 114.48(15), N3–Al1–F101 103.57(14), N4–Al2–N5 102.41(17), N5–Al2–N6 115.61(17), N4–Al2–N6 122.56(17), N4–Al2–F201 102.66(15), N5–Al2–F201 112.48(16), N6–Al2–F201 100.55(16).

Orange crystals suitable for X-ray diffraction analysis were grown directly from the solution of reaction (a) in Scheme 3 and revealed the formation of [CPh_3_]^+^[((C_6_F_5_)_2_N)_3_AlF–Li–FAl(N(C_6_F_5_)_2_)_3_]^–^ (Chart 4). The presence of lithium in the crystal structure can be referred to traces of lithium chloride or Li^+^[ClAl(N(C_6_F_5_)_2_)_3_]^–^ in the used Al[N(C_6_F_5_)_2_]_3_. The selective preparation of [CPh_3_]^+^[((C_6_F_5_)_2_N)_3_AlF–Li–FAl(N(C_6_F_5_)_2_)_3_]^–^ could be achieved from the reaction of two equivalents of *in situ* generated [CPh_3_]^+^[FAl(N(C_6_F_5_)_2_)_3_]^–^ with *n*-butyllithium (Scheme 3(b)). A quartet with a chemical shift of –179.3 ppm and a ^1^*J*(F,Li) coupling constant of 94 Hz is observed for the fluorine atoms bound to aluminum atoms in the ^19^F NMR spectrum. The crystal structure reveals Al–F distances of 1.714(3) and 1.710(3) Å. The lithium cation possesses a distorted octahedral coordination sphere and is coordinated by the two aluminum bound fluorine atoms and additional four *ortho*-fluorine atoms of C_6_F_5_ units. The Li···F distances to the aluminum-bound fluorine atoms are comparably short (1.793(9) and 1.809(9) Å) whereas the distances to the organically bound fluorine atoms range from 2.185(12) to 2.392(10) Å. The R_3_Al–F–Li–F–AlR_3_ structure motive has already been described in the literature for [Ag(PhCH_3_)_3_]^+^[{((SiMe_3_)_3_C)_2_Al_2_F_5_}_2_Li]^–^ (Al–F 1.688(2) Å, Li–F 1.854(6) Å),^53^ [Li(Me_3_Si)_3_CAlF_3_(THF)]_4_ (Al–F 1.694(2) Å and 1.701(2) Å, Li–F 1.873(6) Å and 1.801(6) Å)^54^ and Li^+^[(Me_3_Si)_3_CAlF_3_]^–^·THF (mean Al–F 1.687(8) Å, mean Li–F 1.85(2) Å).^55^ In contrast to [CPh_3_]^+^[((C_6_F_5_)_2_N)_3_AlF–Li–FAl(N(C_6_F_5_)_2_)_3_]^–^, all lithium atoms in these references exhibit a tetrahedral coordination sphere. The three compounds described in the literature show slightly shorter Al–F distances and longer Li–F distances than found for [CPh_3_]^+^[((C_6_F_5_)_2_N)_3_AlF–Li–FAl(N(C_6_F_5_)_2_)_3_]^–^.

### Synthesis and structural features of the metallates

**Scheme 4:**
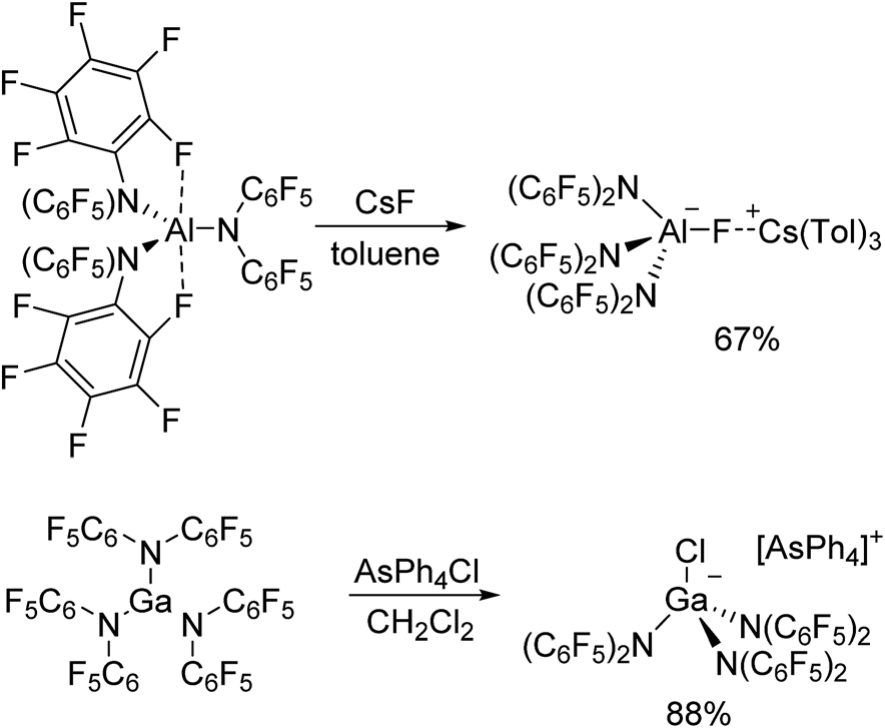
Preparation of [Cs(Tol)_3_]^+^[FAl(N(C_6_F_5_)_2_)_3_]^–^ and [AsPh_4_]^+^[ClGa(N(C_6_F_5_)_2_)_3_]^–^.

**Chart 5:**
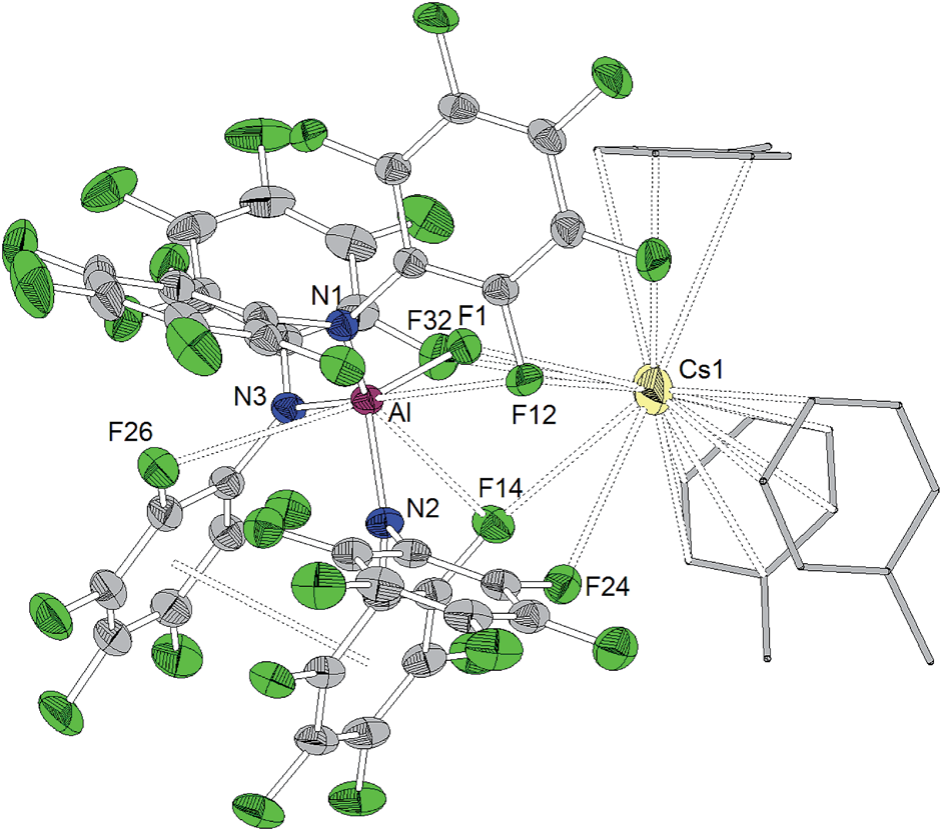
Molecular structure of [Cs(Tol)_3_]^+^[FAl(N(C_6_F_5_)_2_)_3_]^–^ (ellipsoids with 30% probability, the three toluene molecules displayed in wireframe design). Selected bond lengths/Å and angles/°: Al–N1 1.863(3), Al–N2 1.860(3), Al–N3 1.865(3), Al–F1 1.689(2), Al···F12 3.099(3), Al···F14 3.169(3), Al···F26 3.202(3), Cs1···F1 2.878(2), Cs1···F12 3.773(2), Cs1···F14 3.094(3), Cs1···F24 3.807(3), Cs1···F32 3.692(3), N1–Al–N2 112.31(14), N2–Al–N3 102.59(14), N3–Al–N1 123.22(15), N1–Al–F1 98.64(12), N2–Al1–F1 113.94(14), N3–Al1–F1 106.43(14).

**Chart 6:**
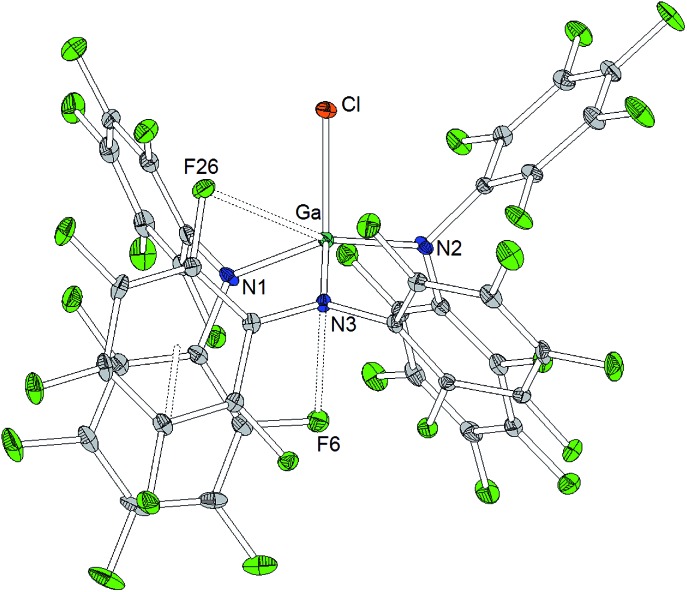
Molecular structure of [AsPh_4_]^+^[ClGa(N(C_6_F_5_)_2_)_3_]^–^ (ellipsoids with 30% probability, the [AsPh_4_]^+^ cation and one pentane molecule are omitted for clarity). Selected bond lengths/Å and angles/°: Ga–N1 1.912(2), Ga–N2 1.912(2), Ga–N3 1.931(2), Ga···F6 3.244(2), Ga···F26 3.205(2), Ga–Cl1 2.1890(8), N1–Ga–N2 117.4(1), N2–Ga–N3 115.7(1), N3–Ga–N1 99.9(0), N1–Ga–Cl1 113.90(8), N2–Ga–Cl1 98.83(7), N3–Ga–Cl1 111.85(7).

The reaction of Al[N(C_6_F_5_)_2_]_3_ with cesium fluoride in toluene and the reaction of Ga[N(C_6_F_5_)_2_]_3_ with tetraphenylarsonium chloride in dichloromethane yielded the two metallates [Cs(Tol)_3_]^+^[FAl(N(C_6_F_5_)_2_)_3_]^–^ and [AsPh_4_]^+^[ClGa(N(C_6_F_5_)_2_)_3_]^–^ (Scheme 4).^56^ The aluminum bound fluorine atom in the former compound exhibits a chemical shift of –157.4 ppm in the ^19^F NMR spectrum. Both metallates could be structurally characterized (Charts 5 and 6, Table 1). The two weakly coordinating anions show the pyramidalization of the coordination geometry around the metal atoms. The M–N bonds are elongated in comparison to the parent compounds whereas this is more pronounced in case of the gallium compound (Al–N between 1.860(3) and 1.865(3) Å, Ga–N between 1.912(2) and 1.931(2) Å). The molecular structure of [Cs(Tol)_3_]^+^[FAl(N(C_6_F_5_)_2_)_3_]^–^ reveals *η*^2^-, *η*^3^- and *η*^6^-coordination of the cesium atom by three toluene molecules and the cesium atom also interacts with the fluorine atom bound to the aluminum center with a Cs···F distance of 2.878(2) Å. Furthermore, the crystal structure reveals four metal–fluorine contacts between the cesium atom and carbon bound fluorine atoms (Cs···F between 3.094(2) and 3.807(3) Å).

**Table 1:** M–N bond lengths and distances between the metal atom and the donor atom in the ate complexes [Cs(Tol)_3_]^+^[FAl(N(C_6_F_5_)_2_)_3_]^–^ and [AsPh_4_]^+^[ClGa(N(C_6_F_5_)_2_)_3_]^–^ in comparison to the free Lewis acids

	Al[N(C_6_F_5_)_2_]_3_	Ga[N(C_6_F_5_)_2_]_3_	[Cs(Tol)_3_]^+^[FAl(N(C_6_F_5_)_2_)_3_]^–^	[AsPh_4_]^+^[ClGa(N(C_6_F_5_)_2_)_3_]^–^
M–N1	1.843(2)	1.826(5)	1.863(3)	1.912(2)
M–N2	1.840(2)	1.798(5)	1.860(3)	1.912(2)
M–N3	1.805(2)	1.848(5)	1.865(3)	1.931(2)
M–Hal	—	—	1.689(2)	2.1890(8)

### Qualitative chloride ion affinity in dichloromethane

**Scheme 5:**
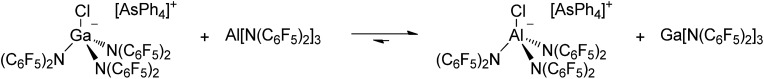
Qualitative competition experiments for the determination of the chloride ion affinity of Al[N(C_6_F_5_)_2_]_3_ and Ga[N(C_6_F_5_)_2_]_3_.

To evaluate the Lewis acidity of Al[N(C_6_F_5_)_2_]_3_ and Ga[N(C_6_F_5_)_2_]_3_ in solution, their chloride ion affinity in dichloromethane was studied by means of qualitative competition experiments *via*^19^F NMR spectroscopy. Both compounds turned out to be able to abstract a chloride ion from [AsPh_4_]^+^[ClB(C_6_F_5_)_3_]^–^. Thus, the borane B(C_6_F_5_)_3_ is a weaker Lewis acid towards a chloride ion in solution than both of the two metal amido title compounds. The addition of one equivalent of tetraphenylarsonium chloride to a 1 : 1 mixture of Al[N(C_6_F_5_)_2_]_3_ and Ga[N(C_6_F_5_)_2_]_3_ resulted in the formation of [AsPh_4_]^+^[ClAl(N(C_6_F_5_)_2_)_3_]^–^ identifying the aluminum complex the stronger Lewis acid under the applied conditions (Scheme 5). Hence, the experimental results for the chloride ion affinity follow the trend of the fluoride ion affinity predicted by theoretical calculations (*vide infra*).

### Theoretical section

As the M[N(C_6_F_5_)_2_]_3_ (M = Al, Ga) Lewis acids are too large for high level *ab initio* calculations, quantum chemical calculations on the Lewis acidities (*i.e.* ion affinities) of M[N(C_6_F_5_)_2_]_3_ (M = Al, Ga) were split into two parts to increase the accuracy. Ligand exchange reactions with their MF_3_ counterparts were calculated at the BP86 57–59-D3 60/def-TZVP^61^ (including Grimme's 2010 dispersion correction) level of theory:
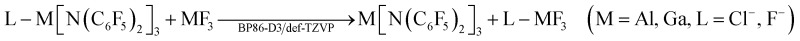


As these reactions are isodesmic, the error in the metal–ligand bond strengths should largely cancel out retaining high accuracy despite of the formally rather low level of theory.

The ligand dissociation energies of the L–MF_3_ complexes were calculated with a CCSD(T)–MP2 compound method based on single point calculations on MP2/def2-QZVPP^62^ structures. Due to the very similar basis set dependency of CCSD(T) and MP2, CCSD(T)/A′VQZ accuracy can be approximated by calculatingΔ*E*_CCSD(T)/A′VQZ_ ≈ Δ*E*_compound_ = Δ*E*_CCSD(T)/A′VDZ_ + Δ*E*_MP2/A′VQZ_ – Δ*E*_MP2/A′VDZ_with A′VXZ = cc-pVXZ for H,^63^ aug-cc-pV(X + d)Z for Al and P,^64^ aug-cc-pwCVXZ-PP for Ga,^65^ aug-cc-pVXZ for 2nd row elements^66^ (X = D, Q).
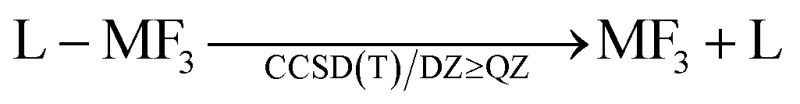


Addition of both reaction energetics gives the M[N(C_6_F_5_)_2_]_3_ (M = Al, Ga) dissociation energies. Thermal corrections to enthalpies and Gibbs energies were done at the BP86-D3/def-TZVP level of theory.

Theoretical calculations reveal an outstanding fluoride ion affinity in the gas phase of 555 kJ mol^–1^ for Al[N(C_6_F_5_)_2_]_3_ (Table 2). This value is practically identical to the FIAs of B(CF_3_)_3_ (556 kJ mol^–1^)^9^ and AuF_5_ (556 kJ mol^–1^).^8^ Thus, Al[N(C_6_F_5_)_2_]_3_ is not only lot more Lewis acidic than the strongest conventional Lewis acid antimony pentafluoride (495 kJ mol^–1^),^9^ but even outnumbers the aluminum-based Lewis acids Al[OC(C_5_F_10_)C_6_F_5_]_3_ (530 kJ mol^–1^)^11^ and Al[OC(CF_3_)_3_]_3_ (543 kJ mol^–1^).^9^ As discussed above, the Lewis acidity of Al[N(C_6_F_5_)_2_]_3_ is reduced by two weak dative bonds from *ortho*-fluorine atoms at the phenyl rings. To evaluate this effect, we calculated the FIA of Al[N(C_6_H_2_F_3_)_2_]_3_ with all *ortho*-fluorine atoms replaced by hydrogen, obtaining an even higher FIA of 598 kJ mol^–1^.

**Table 2:** Calculated ion affinities in the gas phase

	FIA/kJ mol^–1^	Chloride ion affinity/kJ mol^–1^
Al[N(C_6_H_2_F_3_)_2_]_3_	598	—
AuF_5_	556^9^	—
**Al[N(C**_**6**_ **F**_**5**_ **)**_**2**_ **]**_**3**_	**555**	**362**
B(CF_3_)_3_	556^12^	358^12^
Al[OC(CF_3_)_3_]_3_	543^12^	352^12^
SbF_5_	493^12^	341^12^
**Ga[N(C**_**6**_ **F**_**5**_ **)**_**2**_ **]**_**3**_	**472**	**324**
AlF_3_	471^12^	306^12^
B(C_6_F_5_)_3_	452^12^	236^12^
BF_3_	342^12^	146^12^

Ga[N(C_6_F_5_)_2_]_3_ exhibits a computational FIA of 472 kJ mol^–1^. Therefore, it is similarly Lewis acidic as for example monomeric AlF_3_ (482 kJ mol^–1^), but stronger than the widely used B(C_6_F_5_)_3_ (452 kJ mol^–1^).^9^ However, the Lewis acidity of Ga[N(C_6_F_5_)_2_]_3_ is located slightly below the threshold to Lewis superacidity. The lower FIA of Ga[N(C_6_F_5_)_2_]_3_ compared to the aluminum homolog can be referred to the stabilization of the Lewis acidic gallium center by six metal fluorine contacts (Chart 3). The formation of the gallate [FGa(N(C_6_F_5_)_2_)_3_]^–^ after the uptake of a fluoride anion leads to an unfavorable situation due to repulsion of the C_6_F_5_ moieties. In case of isostructural aluminum and gallium centered Lewis acids, a decrease in Lewis acidity is also expected for the gallium compound due to gallium's higher electronegativity as a consequence of the d-block contraction.

The Lewis acids treated herein show chloride ion affinities in the gas phase of 362 kJ mol^–1^ for Al[N(C_6_F_5_)_2_]_3_ and 324 kJ mol^–1^ in case of the corresponding gallium complex. These values are in agreement with the experimental chloride affinities in dichloromethane. They are considerably lower than the corresponding FIA values and the comparison to the chloride affinities of other Lewis acids shows that Al[N(C_6_F_5_)_2_]_3_ and Ga[N(C_6_F_5_)_2_]_3_ are in the same positions as in the FIA ranking.^67^ However, it is striking that the difference between the chloride affinities of Al[N(C_6_F_5_)_2_]_3_ and Ga[N(C_6_F_5_)_2_]_3_ is much smaller than the deviation between the FIA values. This can be attributed to the softer character of chloride in comparison to the hard fluoride anion.

## Conclusions

We presented the first metal amide with a higher fluoride ion affinity in the gas phase and in solution than the benchmark compound for Lewis superacidity SbF_5_. In this context, the synthesis and full characterization of two homoleptic group 13 metal decafluorodiphenylamides Al[N(C_6_F_5_)_2_]_3_ (ALTA) and Ga[N(C_6_F_5_)_2_]_3_ (GATA) are described. The origin for such high fluoride affinity of Al[N(C_6_F_5_)_2_]_3_ is originating from a weak amide donor capability and a trigonal planar AlN_3_ coordination motif with two hemilabile secondary *ortho*-CF → Al contacts. In contrast, the slightly weaker Lewis acid Ga[N(C_6_F_5_)_2_]_3_ has a trigonal pyramidal GaN_3_ configuration with six extra *ortho*-CF → Ga contacts in the solid state. In solution the secondary interactions of both compounds are involved in a fast dynamic exchange process. In contrast to the prominent Lewis superacidic perfluoroalkoxide [Al(OC(CF_3_)_3_)_3_] which has been crystallized as base adducts only, the amide Al[N(C_6_F_5_)_2_]_3_ can be isolated as crystalline Lewis acid. The fluoride affinity is experimentally substantiated by fluoride abstraction from F–X (X = BF_3_, B(C_6_F_5_)_3_, SbF_5_ and CPh_3_^+^) as well as by competition experiments between both title compounds. The prominent status of the Lewis superacid Al[N(C_6_F_5_)_2_]_3_ is emphasized by the calculated fluoride ion affinity (FIA) in the gas phase of 555 kJ mol^–1^*versus* 493 kJ mol^–1^ for SbF_5_.

We believe that these and related Lewis acidic aluminum and gallium amides add new perspectives to the highly topical field of frustrated Lewis pairs that has mainly been dominated by boron-based Lewis acids so far. Furthermore, the weakly coordinating anions derived from our metal amides will be applied for stabilization of highly reactive cations in catalysis and fundamental chemistry.

## Conflicts of interest

There are no conflicts to declare.

## Supplementary Material

Supplementary information

Crystal structure data
