# Supplementary material for: The Lewis superacid Al[N(C6F5)2]3 and its higher homolog Ga[N(C6F5)2]3 – structural features, theoretical investigation and reactions of a metal amide with higher fluoride ion affinity than SbF5
Source: Chem Sci. 2017 Oct 23;9(1):245–53. doi: 10.1039/c7sc03988c (PMC5869307; doi:10.1039/c7sc03988c)
Supplement: Supplementary file 1 [file SC-009-C7SC03988C-s001.pdf]

Electronic Supplementary Material (ESI) for Chemical Science.
This journal is © The Royal Society of Chemistry 2017

Supporting Information

The New Lewis Superacid $Al[N(C_6F_5)_2]_3$ and its Higher Homolog $Ga[N(C_6F_5)_2]_3$ - Structural Features, Theoretical Investigation and Reactions of a Metal Amide with Higher Fluoride Ion Affinity than SbF_5

Julius F. Kögel, Denis A. Sorokin, Alexander Khvorost, Martin Scott, Klaus Harms, Daniel Himmel, Ingo Krossing, Jörg Sundermeyer*

Table of Contents

Synthetic Procedures S2

Crystallographic Information S6

Quantumchemical Calculations S9

References S23

Synthetic Procedures

All reactions were carried out under inert atmosphere using standard Schlenk techniques. Moisture and air sensitive substances were stored in a conventional nitrogen-flushed glovebox. Solvents were purified according to literature procedures and kept under an inert atmosphere. $HN(C_6F_5)_2$ was prepared from the reaction of hexafluorobenzene with lithium amide[1] and deprotonated with *n*-butyllithium to obtain $LiN(C_6F_5)_2$.[2] Commercially available aluminum trichloride was sublimed prior to use.

Spectra were recorded on the following spectrometers: NMR: BRUKER ARX300, BRUKER DRX400, BRUKER, DRX500; IR: ATR-FT-IR; MS: LTQ-FT or QStarPulsar i (FINNIGAN); elemental analysis: CHN-Rapid (HERAEUS).

Preparation of $Al[N(C_6F_5)_2]_3$: A solution of *n*-butyllithium (1.6 M, 8.0 mL, 12.8 mmol, 3.1 eq.) in hexane was added dropwise over 1 min to a solution of $HN(C_6F_5)_2$ (4.48 g, 12.8 mmol, 3.1 eq.) in toluene (45 mL) at -50 °C. The formed suspension of $LiN(C_6F_5)_2$ was stirred for 20 min at -50 °C and a suspension of finely pesteled aluminum trichloride (0.55 g, 4.1 mmol) in toluene (20 mL) was added. The reaction mixture was allowed to reach room temperature and stirred for 18 h at 75 °C. The hot suspension was filtered and large colorless crystals grew from the filtrate after 12 h at room temperature. The crystals were separated from the supernatant by decantation, recrystallized from toluene, washed with pentane and dried *in vacuo* to give $Al[N(C_6F_5)_2]_3$ (2.06 g, 1.9 mmol, 47%) as a white solid. The yield strongly depends on the grain size of the used $AlCl_3$. Single crystals suitable for structure determination were obtained by layering a saturated solution in toluene with pentane at -30 °C.

M. p. 167.8 °C (decomposition); ^{13}C NMR (101 MHz, $[D_6]$benzene, 25 °C, TMS): δ=143.8 (dm, ^1J(C,F) = 240.2 Hz, *o*-C_{Ar}), 138.3 (dm, ^1J(C,F) = 255.4 Hz, *p*-C_{Ar}), 137.8 (dm, ^1J(C,F) = 241.2 Hz, *m*-C_{Ar}), 120.7 ppm (s, *ipso*-C_{Ar}); ^{19}F NMR (188.2 MHz, $[D_6]$benzene, 25 °C, $CFCl_3$): δ=-153.1 (d, ^3J(F,F) = 20 Hz, 12F, *o*-F), -158.6 (t, ^3J(F,F) =21 Hz, 6F, *p*-F), -161.1 ppm (t, ^3J(F,F) = 21 Hz, 12F, *m*-F); IR: $\tilde{\nu}$=440 (m), 453 (w), 507 (w), 619 (w), 638 (w), 683 (m), 764 (m), 802 (br m), 990 (s), 1030 (br s), 1096 (br m), 1182 (w), 1211 (m), 1507 (s), 1520 (s) cm^{-1}; elemental analysis calcd (%) for $C_{36}AlF_{30}N_3$: C 40.36, N 3.92; found: C 39.48, N 3.73.

$Al[N(C_6F_5)_2]_3$ can also be prepared from the reaction of $AlMe_3$ with $HN(C_6F_5)_2$: $AlMe_3$ (0.21 g, 3.10 mmol, 1.0 eq.) was added dropwise to a solution of $HN(C_6F_5)_2$ (3.10 g, 8.89 mmol, 3.0 eq.) in *o*-xylene (25 mL) and the reaction mixture was stirred for 90 h at 130 °C. After allowing the solution to reach room temperature, big brownish crystals precipitated. They were separated from the supernatant, recrystallized from toluene and dried *in vacuo* to give $Al[N(C_6F_5)_2]_3$ (0.31 g, 0.29 mmol, 9%) as a white solid.

The reaction of $Al[N(SiMe_3)_2]_3$ with $HN(C_6F_5)_2$ in toluene at 90 °C did not yield the desired $Al[N(C_6F_5)_2]_3$.

Preparation of $[Cs(Tol)_3]^+[FAl(N(C_6F_5)_2)_3]^-$: $Al[N(C_6F_5)_2]_3$ (0.360 g, 0.33 mmol, 1.0 eq.) and cesium fluoride (0.102 g, 0.66 mmol, 2.0 eq.) were suspended in toluene (10 mL) and the reaction mixture was stirred for 20 h at 100 °C. The hot solution was filtered to separate remaining CsF and the filtrate was cooled to room temperature and set aside for crystallization. Colorless crystals formed that were separated from the supernatant by filtration, washed with pentane and dried in the argon stream to yield $[Cs(Tol)_3]^+[FAl(N(C_6F_5)_2)_3]^-$ (0.330 g, 0.22 mmol, 67%) as a white crystalline solid.
M. p. 124.7 °C; ^{1}H NMR (200 MHz, [D$_6$]benzene, 25 °C, TMS): δ=6.98-7.17 (m, 15H, C_6H_5), 2.10 ppm (s, 9H, CH_3); ^{19}F-NMR (188 MHz, [D$_6$]benzene, 25 °C, $CFCl_3$): δ=-150.4 (br s, 12F, *o*-F), -157.4 (br s, 1F, Al-F), -164.0 (t, ^3J(F,F) = 20 Hz, 6F, *p*-F), -166.3 ppm (t, ^3J(F,F) = 22 Hz, 12F, *m*-F); elemental analysis calcd (%) for $C_{57}H_{24}AlCsF_{31}N_3$: C 45.65, H 1.61, N 2.80; found: C 44.89, H 1.68, N 2.76.

Preparation of $[CPh_3]^+[FAl(N(C_6F_5)_2)_3]^-$: A solution of $[Ph_3C]^+[BF_4]^-$ (0.31 g, 0.94 mmol, 1.0 eq.) in dichloromethane (10 mL) was added dropwise to a suspension of $Al[N(C_6F_5)_2]_3$ (1.00 g, 0.94 mmol, 1.0 eq.) in toluene (30 mL). The reaction mixture changed color to red. A pH indicator paper in the argon stream of the reaction mixture changes color to red indicating the formation of BF_3. All volatiles were removed *in vacuo* and the residue was suspended in toluene (20 mL) and filtrated. Hexane (30 mL) was added to the filtrate leading to the precipitation of a yellow solid. It was filtered off, washed with pentane and dried *in vacuo*. It decomposes after 24 h, but is stable for at least three days in toluene solution. A NMR spectroscopically identical compound could be obtained directly from the reaction between $Al[N(C_6F_5)_2]_3$ and trityl fluoride in C_6D_6. ^{1}H NMR (200 MHz, [D$_6$]benzene, 25 °C, TMS): δ=7.38 (t, ^3J(H,H) = 7.5 Hz, 3H, C_6H_5), 7.08 (t, ^3J(H,H) = 7.8 Hz, 6H, C_6H_5), 6.78 ppm (d, ^3J(H,H) = 7.5 Hz, 6H, C_6H_5); ^{19}F NMR (188 MHz, [D$_6$]benzene, 25 °C, $CFCl_3$): δ=-148.8 (d, ^3J(F,F) = 22 Hz, 12F, *o*-F), -164.8 (t, ^3J(F,F) = 22 Hz, 6F, *p*-F), -166.3 (t, ^3J(F,F) = 21 Hz, 12F, *m*-F), -170.8 ppm (br s, 1F, Al-F).

Reaction of $Al[N(C_6F_5)_2]_3$ with $[Ph_4P]^+[SbF_6]^-$: A solution of $[Ph_4P]^+[SbF_6]^-$ (75 mg, 0.13 mmol, 1.0 eq.) in toluene (1 mL) was added dropwise to a suspension of $Al[N(C_6F_5)_2]_3$ (140 mg, 0.13 mmol, 1.0 eq.) in toluene (2 mL). The reaction mixture was slowly heated under dissolution of $Al[N(C_6F_5)_2]_3$. A color change to yellow was observed at 100 °C and a white precipitate was formed. It was separated by filtration and dried *in vacuo*. A ^{19}F NMR spectrum was measured: ^{19}F NMR (188 MHz, [D$_6$]benzene, 25 °C, $CFCl_3$): δ=-149.6 ppm (br s, ^3J(F,F) = 22 Hz, 12F, *o*-F), -166.2 (t, ^3J(F,F) = 22 Hz, 6F, *p*-F), -167.5 (t, ^3J(F,F) = 21 Hz, 12F, *m*-F), -172.3 (br s, 1F, Al-F).

Reaction of $Al[N(C_6F_5)_2]_3$ with $[Ph_3C]^+[FB(C_6F_5)_3]^-$: $[Ph_3C]^+[FB(C_6F_5)_3]^-$ (10 mg, 0.013 mmol, 1 eq.) was added to a solution of $Al[N(C_6F_5)_2]_3$ (15 mg, 0.014 mmol, 1.1 eq.) in [D$_6$]benzene (0.7 mL). The yellow reaction mixture was heated to 80 °C and changed color to yellowish brown under dissolution of $Al[N(C_6F_5)_2]_3$. The ^{19}F NMR spectrum revealed the signals of $B(C_6F_5)_3$, $[FAl(N(C_6F_5)_2)_3]^-$ and excess

$Al[N(C_6F_5)_2]_3$. ^{19}F NMR (188 MHz, [D$_6$]benzene, 25 °C, $CFCl_3$): $[FAl(N(C_6F_5)_2)_3]^-$: δ=-170.8 (br s, 1F, Al-F), -166.3 (t, ^3J(F,F) = 21 Hz, 12F, *m*-F), -164.8 (t, ^3J(F,F) = 22 Hz, 6F, *p*-F), -148.8 ppm (d, ^3J(F,F) = 22 Hz, 12F, *o*-F); $B(C_6F_5)_3$: δ=-160.3 (t, ^3J(F,F) = 22 Hz, 6F, *m*-F), -141.8 (t, ^3J(F,F) = 20 Hz, 3F, *p*-F), -129.1 ppm (d, ^3J(F,F) = 22 Hz, 6F, *o*-F).

Reaction of $Al[N(C_6F_5)_2]_3$ with $F_2ClC\text{-}CCl_2F$: $F_2ClC\text{-}CCl_2F$ (2 mL, freshly distilled from P_4O_{10}) was added to $Al[N(C_6F_5)_2]_3$ (100 mg, 0.1 mmol). The reaction mixture was stirred for 5 min at 50 °C. The solution turned dark and a black solid precipitated indicating decomposition of the reactant.

Reaction of $Al[N(C_6F_5)_2]_3$ with C_6F_6: C_6F_6 (2 mL, freshly distilled from P_4O_{10}) was added to $Al[N(C_6F_5)_2]_3$ (100 mg, 0.1 mmol). The reaction mixture was stirred for 5 min at 60 °C. The ^{19}F NMR spectrum of the solution did not show any reaction. A black precipitate formed after stirring the reaction mixture for 2 h at 65 °C indicating decomposition of the reactant.

Preparation of $[CPh_3]^+[((C_6F_5)_2N)_3AlF\text{-}Li\text{-}FAl(N(C_6F_5)_2)_3]^-$: A solution of *n*-butyllithium in hexane (0.200 mmol, 2.0 eq.) was added dropwise to a solution of *in situ* generated $[CPh_3]^+[FAl(N(C_6F_5)_2)_3]^-$ (533.5 mg, 0.400 mmol, 1.0 eq.) in toluene (10 mL) at -80 °C. The red solution changed color to orange and the reaction mixture was allowed to slowly reach room temperature. Cooling the solution again led to the precipitation of orange crystals that were collected and dried *in vacuo*. $[CPh_3]^+[((C_6F_5)_2N)_3AlF\text{-}Li\text{-}FAl(N(C_6F_5)_2)_3]^-$ (287 mg, 0.118 mmol, 59%) was obtained as an orange crystalline solid. M. p. 195.7 °C; ^1H NMR (200 MHz, [D$_6$]benzene, 25 °C, TMS): δ=7.14 (t, ^3J(H,H) = 7.5 Hz, 1H, C_6H_5), 7.04 (t, ^3J(H,H) = 7.8 Hz, 2H, C_6H_5), 6.94 ppm (d, ^3J(H,H) = 7.5 Hz, 2H, C_6H_5); ^{19}F-NMR (188 MHz, [D$_6$]benzene, 25 °C, $CFCl_3$): δ=-149.6 (br s, 24F, *o*-F), -162.3 (t, ^3J(F,F) = 22 Hz, 12F, *p*-F), -164.2 (t, 24F, ^3J(F,F) = 21 Hz, *m*-F), -179.3 ppm (q, ^1J(F,Li) = 94 Hz, 2F, Al-F); elemental analysis calcd (%) for $C_{91}H_{15}Al_2F_{62}LiN_6$: C 44.96, H 0.62, N 3.46; found: C 44.53, H 0.65, N 3.34.

Preparation of $[Cp_2ZrMe]^+[FAl(N(C_6F_5)_2)_3]^-$: A solution of Cp_2ZrMe_2 (39 mg, 0.16 mmol, 1.0 eq.) in toluene (3 mL) was added dropwise to a *in situ* generated solution of $[CPh_3]^+[FAl(N(C_6F_5)_2)_3]^-$ (210 mg, 0.16 mmol, 1.0 eq.) in toluene (7 mL) at -50 °C. The reaction mixture decolorized, was warmed to room temperature and stirred at that temperature for 1 h. All volatiles were removed *in vacuo* and the residue was washed with pentane and recrystallized from hot benzene. After drying *in vacuo*, $[Cp_2ZrMe]^+[FAl(N(C_6F_5)_2)_3]^-$ (153 mg, 0.12 mmol, 73%) was obtained as a white crystalline solid.

M. p. 107.5 °C (decomp.); ^1H NMR (200 MHz, [D$_6$]benzene, 25 °C, TMS): δ=5.81 (s, 10H, C_5H_5), 0.12 ppm (br s, 3H, Zr-Me); ^{19}F-NMR (188 MHz, [D$_6$]benzene, 25 °C, $CFCl_3$): δ=-148.1 (br s, 1F, AlF), -149.4 (br s, 12F, *o*-F), -160.6 (t, ^3J(F,F) = 22 Hz, 6F, *p*-CF), -163.5 ppm (t, ^3J(F,F) = 20 Hz, 12F, *m*-F); elemental analysis calcd (%) for $C_{47}H_{13}AlF_{31}N_3Zr$: C 42.55, H 0.99, N 3.17; found: C 41.76, H 1.08, N 3.31.

Preparation of $Ga[N(C_6F_5)_2]_3$: A solution of gallium trichloride (0.330 g, 1.878 mmol, 1.0 eq.) in toluene (10 mL) was added to a suspension of $LiN(C_6F_5)_2$ (2.000 g, 5.633 mmol, 3.0 eq.) in toluene (40 mL). The reaction mixture was stirred for 16 h at 90 °C and the hot suspension was filtered over Celite®. The filter cake was extracted twice with toluene (20 mL) and the filtrate was evaporated to dryness *in vacuo*. The white residue was washed twice with hexane (40 mL). After drying *in vacuo* $Ga[N(C_6F_5)_2]_3$ (1.120 g, 1.005 mmol, 54%) was obtained as a white solid. Single crystals suitable for structure determination were obtained by cooling a saturated solution in toluene from 90 °C to -30 °C.
^{13}C NMR (126 MHz, [D$_6$]benzene, 25 °C, TMS): δ=142.8 (dd, ^1J(C,F) = 246.5 Hz, ^2J(C,F) = 11.5 Hz, *o*-C_{Ar}), 138.9 (dt, ^1J(C,F) = 256.3 Hz, ^2J(C,F) = 13.5 Hz, *p*-C_{Ar}), 137.9 (dt, ^1J(C,F) = 256.3 Hz, ^2J(C,F) = 12.9 Hz, *m*-C_{Ar}), 120.8 ppm (t, ^2J(C,F) = 9.3 Hz, *ipso*-C_{Ar}); ^{19}F NMR (376 MHz, [D$_6$]benzene, 25 °C, $CFCl_3$): δ=-151.8 (d, ^3J(F,F) = 20.6 Hz, 12F, *o*-F), -158.1 (dt, ^3J(F,F) = 21.7 Hz, ^4J(F,F) = 4.8 Hz, 6F, *p*-F), -161.4 ppm (t, ^3J(F,F) = 21.1 Hz, 12F, *m*-F); IR: $\tilde{\nu}$=410 (w), 498 (w), 573 (w), 639 (m), 669 (w), 712 (w), 725 (w), 806 (w), 837 (m), 987 (s), 1026 (s), 1140 (w), 1191 (w), 1264 (w), 1322 (w), 1369 (w), 1501 (s), 1632 (w) cm^{-1}; elemental analysis calcd (%) for $C_{36}F_{30}GaN_3$: C 38.81, N 3.77; found: C 38.21, N 3.67.

Preparation of $[AsPh_4]^+[ClGa(N(C_6F_5)_2)_3]^-$: A solution of $Ga[N(C_6F_5)_2]_3$ (200 mg, 0.180 mmol, 1.0 eq.) in dichloromethane (7.5 mL) was added to a solution of tetraphenylarsonium chloride (75 mg, 0.180 mmol, 1.0 eq.) in dichloromethane (7.5 mL). The reaction mixture was stirred for 1 h at room temperature and evaporated to dryness *in vacuo*. The remaining solid was washed with pentane (15 mL) and dried *in vacuo*. $[AsPh_4]^+[ClGa(N(C_6F_5)_2)_3]^-$ (242 mg, 0.158 mmol, 88%) was obtained as a white solid. Single crystals suitable for structure determination were obtained by layering a saturated solution in diethylether with pentane at -30 °C. ^1H NMR (400 MHz, CD_2Cl_2, 25 °C, TMS): δ=7.87 (t, ^3J(H,H) = 7.5 Hz, 4H, *p*-H), 7.75 (t, ^3J(H,H) = 7.8 Hz, 8H, *m*-H), 7.61 ppm (d, ^3J(H,H) = 7.5 Hz, 8H, *o*-H); ^{13}C NMR (101 MHz, CD_2Cl_2, 25 °C, TMS): δ=145.6 (dm, ^1J(C,F) = 245.2 Hz, *p*-C), 139.1 (dm, ^1J(C,F) = 247.2 Hz, CF_{Ar}), 135.4 (*p*-C_{Ph}), 133.3 (C_{Ph}), 131.7 (C_{Ph}), 126.6 (*ipso*-C), 120.8 ppm (*ipso*-C_{Ph}) (the remaining carbon atoms belonging to the C_6F_5 groups were not detected due to signal overlay); ^{19}F NMR (282 MHz, CD_2Cl_2, 25 °C, $CFCl_3$): δ=-147.7 (d, ^3J(F,F) = 18.1 Hz, 6F, F_{Ar}), -165.4 (t, ^3J(F,F) = 21.7 Hz, 3F, *p*-F), -167.0 ppm (t, ^3J(F,F) = 19.9 Hz, 6F, F_{Ar}); ESI-MS (CH_2Cl_2): *m*/*z* (%): 383 (100) $[M]^+$, HRMS (ESI): *m*/*z* calcd for $C_{24}H_{20}As^+$: 383.0775 $[M]^+$; found: 383.0774, (-)-ESI-MS (CH_2Cl_2): *m*/*z* (%): 348 (100) $[N(C_6F_5)_2]^-$, (-)-HRMS (ESI): *m*/*z* calcd for $C_{36}ClF_{30}GaN_3^-$: 1147.8563 $[M]^+$; found: 1147.8576; elemental analysis calcd (%) for $C_{60}H_{20}AsClF_{30}GaN_3$: C 47.01, H 1.32, N 2.74; found: C 47.17, H 1.79, N 2.83.

Crystallographic Information

IPDS I, IPDS II (Stoe) and D8 QUEST (Bruker) diffractometers were used for data collection by the x-ray department at the Philipps-Universität Marburg (*Dr. K. Harms*, *G. Geiseler*, *M. Marsch*, and *R. Riedel*). Data collection, reduction and cell refinement was performed with Stoe IPDS Software or Apex2 (Bruker). Structures were solved with SIR92,[3] SIR97,[4] SIR2004[5] or SHELXS-97[6] and refined with SHELXL-97.[7] Absorption correction was performed with semi-empirical methods within WinGX (Multi-scan[8] or Gaussian[9]) or Apex2 (Multi-scan[10]).

Hydrogen atoms were calculated in their idealized positions and refined with fixed isotropic thermal parameters. All molecular structures were illustrated with Diamond 3[11] using thermal ellipsoids at the 30% probability level for all non-hydrogen atoms.

Crystal data and experimental conditions are listed in S-Table S1-S3. The corresponding CIF files providing full information concerning the molecular structures and experimental conditions are deposited at the Cambridge Crystallographic Data Center.

Table S1. Crystal data and experimental conditions.

	**$Al[N(C_6F_5)_2]_3$**	**$[Cs(PhMe)_3]^+$ $[FAl(N(C_6F_5)_2)_3]^-$**	**$[CPh_3]^+$ $[Li(FAl(N(C_6F_5)_2)_3)_2]^-$**
empirical formula	$C_{36}AlF_{30}N_3$	$C_{57}H_{24}AlCsF_{31}N_3$	$C_{91}H_{15}Al_2F_{62}LiN_6$, $2(C_7H_8)$
mol. weight [g mol^{-1}]	1071.37	1499.68	2615.26
crystal habit	colorless prism	colorless, irregular	colorless prism
crystal size [mm^3]	0.66 · 0.24 · 0.12	0.54 · 0.39 · 0.11	0.41 · 0.34 · 0.28
crystal system	triclinic	monoclinic	triclinic
space group	*P*-1	*P*121/*c*1	*P*-1
a [Å]	10.8600(10)	13.0294(5)	14.4989(14)
b [Å]	12.7006(11)	26.0373(7)	18.051(2)
c [Å]	14.3671(13)	16.8481(6)	21.909(2)
α [°]	73.501(10)	90	84.427(13)
β [°]	72.779(11)	92.421(3)	73.249(12)
γ [°]	89.315(11)	90	67.033(12)
volume [Å^3]	1809.2(3)	5710.6(3)	5054.8(9)
Z	2	4	2
density [g cm^{-3}]	1.967	1.744	1.718
T [K]	193(2)	193(2)	193(2)
absorption coeff. [mm^{-1}]	0.248	0.808	0.198
Θ range [°]	2.0 to 25.9	1.4 to 26.0	1.9 to 25.0
index ranges	-13 <= h <= 13, -15 <= k <= 15, -17 <= l <= 17	-15 <= h <= 14, -31 <= k <= 31, -20 <= l <= 20	-17 <= h <= 17, -21 <= k <= 21, -26 <= l <= 26
reflns collected	17179	44417	45908
independent reflns	6574 [R(int) = 0.0444]	11041 [R(int) = 0.0308]	16752 [R(int) = 0.0581]
absorption correction	empirical	multi-scan	gaussian
max. and min. transmission	0.931 and 0.971	0.8482 and 0.6968	0.9551 and 0.932
transmissiondata/restraints /parameters	6574 / 1 / 631	11041 / 0 / 802	16752 / 11 / 1458
goodness-of-fit on F^2	0.853	1.097	0.793
final R indices [I>2s(I)]	R1 = 0.0375	R1 = 0.0601	R1 = 0.0538
R indices (all data)	wR2 = 0.0914	wR2 = 0.1779	wR2 = 0.1483
larg. diff. peak/hole [e Å^{-3}]	0.274 / -0.308	1.245 / -2.580	0.764 / -0.701
treatment of H atoms	-	constr.	constr.

Table S2. Crystal data and experimental conditions.

	$Ga[N(C_6F_5)_2]_3$	$[AsPh_4]^+$ $[ClGa(N(C_6F_5)_2)_3]^-$
empirical formula	$C_{36}F_{30}GaN_3$	$C_{120}H_{40}As_2Cl_2F_{60}Ga_2N_6$, C_5H_{12}
mol. weight [g mol^{-1}]	1114.11	3137.91
crystal habit	colorless prism	colorless plate
crystal size [mm^3]	0.30 · 0.05 · 0.04	0.28 · 0.08 · 0.05
crystal system	monoclinic	triclinic
space group	*P*21/*c*	*P*-1
a [Å]	7.9908(7)	11.2887(5)
b [Å]	20.611(3)	13.9044(7)
c [Å]	22.8236(19)	20.9274(11)
α [°]	90	98.126(2)
β [°]	108.292(7)	103.279(2)
γ [°]	90	111.8210(10)
volume [Å^3]	3569.1(7)	2873.0(2)
Z	4	1
density [g cm^{-3}]	2.073	1.802
T [K]	100(2)	100(2)
absorption coeff. [mm^{-1}]	0.966	1.241
Θ range [°]	1.4 to 26.9	2.0 to 27.0
index ranges	-10 <= h <= 9, -24 <= k <= 26, -28 <= l <= 28	-14 <= h <= 14, -17 <= k <= 17, -26 <= l <= 26
reflns collected	19281	55879
independent reflns	7542 [R(int) = 0.1328]	12537 [R(int) = 0.0897]
absorption correction	multi-scan	multi-scan
max. and min. transmission	0.9624 and 0.7604	1.0000 and 0.8474
transmissiondata/restraints /parameters	7542 / 0 / 625	12537 / 30 / 913
goodness-of-fit on F^2	0.712	1.074
final R indices [I>2s(I)]	R1 = 0.0509	R1 = 0.0375
R indices (all data)	wR2 = 0.1359	wR2 = 0.1042
larg. diff. peak/hole [e Å^{-3}]	0.991 / -1.138	0.662 / -0.649
treatment of H atoms	-	constr.

Quantumchemical Calculations

DFT optimizations were carried out with TURBOMOLE[12,13] at the (RI-)BP86[14,15,16]/def-TZVP[17], with RI-J auxiliary bases[18,19,20] and D3[21] dispersion correction.Vibrational frequencies were calculated analytically with the AOFORCE[22,23] module and all structures represented true minima without imaginary frequencies on the respective hypersurface. Thermal contributions to *ab initio* reaction energies (see below) were calculated with inclusion of zero point energy, thermal contributions to the enthalpy/entropy (FREEH tool; unscaled[24] BP86/def-TZVP/D3 vibrational frequencies).

RI-MP2 structure optimisations were carried out with TURBOMOLE[12] and def2-QZVPP[17] basis sets and corresponding RI-C auxiliary bases[14] for all atoms. Frozen core: 1s for 2nd row elements, 1s2s2p for Al, none for H, 1s2s2p3s3p for Ga.

MP2 and CCSD(T) single point calculations with correlation-consistent basis sets were done with Gaussian 03.[13] Basis set extrapolation was done with the following formula:

$$\Delta E_{CCSD(T)/A'VQZ} \approx \Delta E_{compound} = \Delta E_{CCSD(T)/A'VDZ} + \Delta E_{MP2/A'VQZ} - \Delta E_{MP2/A'VDZ}$$

with A'VXZ = cc-pVXZ for H[25], aug-cc-pV(X+d)Z for Al[26], aug-cc-pwCVXZ-PP for Ga[27], aug-cc-pVXZ for 2nd row elements [28] (X = D, Q).

Frozen core: 1s for 2nd row elements, 1s2s2p for Al, none for H and Ga.

Thermal contributions to reaction energies were calculated with BP86-D3/def-TZVP at 1 bar, 298.15 K:

$\Delta U° = \Delta E_{QM.} + \Delta E_{vrt}$

($\Delta E_{QM} = \Delta E_{SCF}$ for DFT, $\Delta E_{compound}$ for ab-initio calculations; ΔE_{vrt} = sum of translational, rotational, and vibrational energy incl. zero point vibrational energy)

$\Delta H° = \Delta U° + \Delta n\, RT$ (Δn = particle number change in reaction equation)

$\Delta G° = \Delta H° - 298.15\ \mathrm{K} \cdot \Delta S°$

Table S3. Details on ab initio calculations (with thermal corrections at BP86-D3/def-TZVP level).

	**CCSD(T)/A'VDZ [H]**	**MP2/A'VDZ [H]**	**MP2/A'VQZ [H]**	**E_{comp} [kJ mol^{-1}]**	**H° [kJ mol^{-1}]**	**G° [kJ mol^{-1}]**
F^-	-99.668634	-99.665948	-99.774407	-348070.66	-347943.15	-348015.74
AlF_3	-541.216273	-541.198926	-541.556053	-1421900.96	-1421867.26	-1421950.52
AlF_4^-	-641.066362	-641.043002	-641.511473	-1684349.70	-1684307.74	-1684396.96
GaF_3	-557.728995	-557.717825	-558.354450	-1465988.94	-1465957.94	-1466045.63
GaF_4^-	-657.566082	-657.549237	-658.296248	-1728401.03	-1728362.13	-1728456.65

Table S4. Details on BP86-D3/def-TZVP calculations.

	**SCF [H]**	**E_{vrt} [kJ mol^{-1}]**	**S° [kJ mol^{-1} K^{-1}]**	**H° [kJ mol^{-1}]**	**G° [kJ mol^{-1}]**
F^-	-99.879116	3.72	0.14547	-262226.42	-262269.79
AlF_3	-542.338235	31.22	0.27925	-1423875.34	-1423958.59
AlF_4^-	-642.410764	39.48	0.29923	-1686607.50	-1686696.72
GaF_3	-2224.913325	28.52	0.29413	-5841478.94	-5841566.63
GaF_4^-	-2324.972266	36.42	0.31705	-6104175.78	-6104270.31
$Al(N(C_6F_5)_2)_3$	-4775.691260	965.88	1.49104	-12537609.04	-12538053.60
$FAl(N(C_6F_5)_2)_3^-$	-4875.791366	973.64	1.49255	-12800414.11	-12800859.12
$Ga(N(C_6F_5)_2)_3$	-6458.327682	966.81	1.48051	-16955370.04	-16955811.45
$FGa(N(C_6F_5)_2)_3^-$	-6558.396066	972.77	1.47152	-17218093.62	-17218532.35
$Al(N(C_6F_5)_2)_3)$	-3584.411955	1185.68	1.26100	-9409685.43	-9410061.39
$FAl(N(N(C_6F_5)_2)_3^-$	-3684.528095	1192.54	1.29373	-9672533.49	-9672919.22

MP2/def2-QZVPP coordinates (atomic units, Turbomole format)

AlF_3, D_{3h}

```
 0.00000000000000   0.00000000000000   0.00000000000000  al
 1.54642228740784  -2.67848197174726   0.00000000000000  f
 1.54642228740784   2.67848197174726   0.00000000000000  f
-3.09284457481566   0.00000000000000   0.00000000000000  f
```

AlF_4^-, T_d

```
 0.00000000000000   0.00000000000000   0.00000000000000  al
 1.84949633922621  -1.84949633922621   1.84949633922621  f
-1.84949633922621   1.84949633922621   1.84949633922621  f
-1.84949633922621  -1.84949633922621  -1.84949633922621  f
 1.84949633922621   1.84949633922621  -1.84949633922621  f
```

GaF_3, D_{3h}

```
 0.00000000000000   0.00000000000000   0.00000000000000  ga
 1.61953241012213  -2.80511241883599   0.00000000000000  f
 1.61953241012213   2.80511241883599   0.00000000000000  f
-3.23906482024423   0.00000000000000   0.00000000000000  f
```

GaF_4^-, T_d

```
 0.00000000000000   0.00000000000000   0.00000000000000  ga
 1.93854254827746  -1.93854254827746   1.93854254827746  f
-1.93854254827746   1.93854254827746   1.93854254827746  f
-1.93854254827746  -1.93854254827746  -1.93854254827746  f
 1.93854254827746   1.93854254827746  -1.93854254827746  f
```

BP86-D3/def-TZVP coordinates (atomic units, Turbomole format)

AlF_3, D_{3h}

0.00000000000000	0.00000000000000	0.00000000000000	al
1.56940806082359	-2.71829449915461	0.00000000000000	f
1.56940806082359	2.71829449915461	0.00000000000000	f
-3.13881612164718	0.00000000000000	0.00000000000000	f

AlF_4^-, T_d

0.00000000000000	0.00000000000000	0.00000000000000	al
1.87974602471639	-1.87974602471639	1.87974602471639	f
-1.87974602471639	1.87974602471639	1.87974602471639	f
-1.87974602471639	-1.87974602471639	-1.87974602471639	f
1.87974602471639	1.87974602471639	-1.87974602471639	f

GaF_3, D_{3h}

0.00000000000000	0.00000000000000	0.00000000000000	ga
1.65443129382760	-2.86555905854132	0.00000000000000	f
1.65443129382760	2.86555905854132	0.00000000000000	f
-3.30886258765519	0.00000000000000	0.00000000000000	f

GaF_4^-, T_d

0.00000000000000	0.00000000000000	0.00000000000000	ga
1.98100527642910	-1.98100527642910	1.98100527642910	f
-1.98100527642910	1.98100527642910	1.98100527642910	f
-1.98100527642910	-1.98100527642910	-1.98100527642910	f
1.98100527642910	1.98100527642910	-1.98100527642910	f

$Al(N(C_6F_5)_2)_3$, C_1

0.06751439987584	0.12896607972366	0.06448722572172	al
1.26114557924050	-0.67476825710019	3.28832927470843	n
-0.18719906172103	-2.34673919239267	-2.44511856762995	n
-0.90671112891604	3.37550087863069	-0.60708293849819	n
-0.25452995655689	-2.02369513823070	5.03933421607934	c
-2.21552205736257	-0.81369644318966	6.34709602401144	c
-3.83452573397034	-2.13511316524962	7.95645684828362	c
-3.49211631692481	-4.73194898882164	8.30664039792928	c
-1.54445545768759	-5.98781477149522	7.03328008094384	c

0.02910747613855	-4.63658673421821	5.40585817181277	c
3.86943709565593	-0.55193076192771	3.65088265034202	c
5.23315871063070	-0.97486552590248	5.89574664814126	c
7.86819431926871	-0.75964348226433	5.96691643834010	c
9.24289142325106	-0.04244178182685	3.82626184971696	c
7.94656710166230	0.44304477266218	1.57235692964254	c
5.34533199431155	0.18506706561897	1.58308862487729	c
1.66564379163984	-2.68924847388435	-4.35403523021411	c
4.01960545249688	-3.76872630429153	-3.78865406151341	c
5.90946251620673	-4.01574935379875	-5.61134242616736	c
5.43955652319794	-3.22244849273128	-8.08845461631019	c
3.09246106410377	-2.18061858118089	-8.71805118048475	c
1.24205981468959	-1.92488753693519	-6.85700710789153	c
-2.30353716725376	-3.91126446701576	-2.36671955794315	c
-2.84500911207734	-6.05020076505986	-3.85871956159386	c
-5.07171200965031	-7.43872409328608	-3.52001948649645	c
-6.82619374853387	-6.79462907911670	-1.65094748885815	c
-6.34427582366043	-4.69566249748017	-0.11591148952313	c
-4.12779466009152	-3.36741637345627	-0.52353545019049	c
-1.46666161452717	4.24081250497874	-3.08043587974737	c
-3.41759555874901	3.19378009566404	-4.54204737991560	c
-3.93170295925350	4.02759887193449	-6.99056160829455	c
-2.51878227782077	6.00408527604463	-8.02307305696860	c
-0.58158835102694	7.11228955338050	-6.60501854051113	c
-0.06724256292821	6.21894908513685	-4.17797872800549	c
-1.25443758482355	5.15994514027292	1.36379156087519	c
-3.65564288425623	6.17672646075400	1.87797200966661	c
-4.05871002063578	7.87551193981028	3.85350132082068	c
-2.03963879147727	8.57301823467270	5.41006276207126	c
0.36635722719442	7.58480536561524	4.95514279219533	c
0.73719427720226	5.93112227371461	2.93515444796662	c
-2.58674648841316	1.68515521792809	6.01552551297914	f
-5.70450602990902	-0.92808694142972	9.17107144254650	f
-5.02834187573567	-6.01729011005490	9.85660921247152	f
-1.22165407708294	-8.48308223223622	7.35992859755187	f
1.87173830242983	-5.87134528465447	4.15787419564950	f
4.02051406878363	-1.60496922971211	8.05054955048278	f
9.09319373287745	-1.21137248587658	8.13997671502691	f
11.76784219016016	0.17282876779094	3.92545191744064	f

9.18256825053870	1.13171028644037	-0.52888902498683	f
3.99571438396326	0.68251778092117	-0.63480683612480	f
4.48859040043811	-4.57340581377513	-1.41699968370707	f
8.15697311685884	-5.02347352196894	-5.00678171180755	f
7.23528920549483	-3.45987014595555	-9.85696290112296	f
2.63411461721482	-1.43835565444643	-11.09928805879517	f
-1.02644582880823	-0.97281606939729	-7.50256503825337	f
-1.22297654833394	-6.82824634741861	-5.66890117688362	f
-5.50942376164947	-9.45356787190735	-4.99391213225957	f
-8.93837715122354	-8.16164048027567	-1.33394929412549	f
-7.96175154420138	-4.00537212008128	1.70737593757698	f
-3.60434494595913	-1.25675897984220	0.97280727524132	f
-4.84865785957300	1.31494005265298	-3.58303219320586	f
-5.78823622818185	2.95265992312230	-8.34581373593747	f
-3.00318797374704	6.82518403330215	-10.37182234571437	f
0.79587062047474	8.99804400545695	-7.60231516131639	f
1.83405625634564	7.27958794807529	-2.85788469202895	f
-5.64224181855026	5.45732487666144	0.45725117157137	f
-6.38010345735764	8.79902209066942	4.30404496675461	f
-2.41250709693337	10.18114406571959	7.33414068419226	f
2.31774058315321	8.26397471029221	6.43240404007721	f
3.09519303006443	5.05315619224130	2.51126684931763	f

$FAl(N(C_6F_5)_2)_3^-$, C_1

-0.49237691634568	-1.61299410488604	-0.38483340821645	al
-1.40353721963105	-4.63197694594500	-1.02384533574972	f
3.68772771267369	2.45984847296226	-6.28421777333642	f
4.52567109600297	0.73341667995492	-11.02078557656299	f
4.39721772505368	-4.36210753439389	-12.01626114992226	f
3.54024065951921	-7.70691199340578	-8.14816820756238	f
2.89473565622627	-6.02444141070904	-3.37917756456209	f
1.43274807242295	4.21262044050629	-0.67230938676732	f
4.97798266363349	7.20490746257831	1.50081815453078	f
9.87494121213658	5.54120139589632	1.96581694337193	f
11.14318606501271	0.80139269936533	0.27775321643534	f
7.57365291908959	-2.25891100518370	-1.76809942735766	f
-6.57277140233547	-0.16082350577355	-5.62437969428286	f
-10.78705204038993	-3.03519726572130	-5.98062379962851	f
-12.25190646765333	-6.05457529796346	-2.01544480846462	f

-9.45289354384851	-6.07146165702872	2.35982458280100	f
-5.33383971854177	-3.07389301156270	2.80402602466528	f
-6.92346643360817	3.10615016091259	1.56643301340800	f
-7.66090414790250	8.06591963977478	0.40735077295236	f
-5.16825885953081	10.19487089183705	-3.61248451230780	f
-2.00551496930603	7.28783351826883	-6.52005219968372	f
-1.38972249543143	2.29908377682567	-5.44742731762012	f
-1.78810052203839	3.84038572943575	3.31906965151625	f
-5.24177335688482	4.91931915614698	6.94593331381027	f
-6.31579490800078	1.44332925160036	10.64273294654295	f
-3.90939489113737	-3.15050679911007	10.63757423007725	f
-0.55667107039969	-4.30004117842912	6.91017562876077	f
3.08875116107537	-5.57451442420498	2.03168986886958	f
7.96856458634911	-6.34684419832179	3.38054571709551	f
10.56657965448369	-2.70930874519441	6.01317911442880	f
8.20640230325089	1.75081759819668	7.23310252068443	f
3.37201764184320	2.60145422208156	5.77272781809461	f
2.49194764066941	-0.77203964536750	-2.22864416977198	n
-3.60643338357389	0.02637847537439	-1.17442131329774	n
0.57839180540803	-0.94810690607014	2.98428414102589	n
3.01714650614540	-1.70535765766097	-4.67126405870812	c
3.58469165912836	-0.05623404307196	-6.69034062591786	c
4.04668309504482	-0.92048168996979	-9.13686471582808	c
3.98292922468108	-3.50759707235241	-9.64832295875935	c
3.49666434885486	-5.19862592927950	-7.68505478651385	c
3.09943672756372	-4.31070510172746	-5.22936582681261	c
4.35643877723856	0.85568637283718	-1.25411074439886	c
3.79329636822908	3.30398650175105	-0.39226549767568	c
5.60651148842888	4.86962300954671	0.70707880398547	c
8.10223204170503	4.04029924227807	0.92015452612340	c
8.74681071817561	1.63903794788708	0.03147328058216	c
6.89761026407720	0.08860393230206	-1.02530112438010	c
-5.79214676514972	-1.48642604893649	-1.37937773475050	c
-7.27379745588690	-1.54342702655865	-3.59343701944073	c
-9.43713329704239	-3.03501420198019	-3.81285792296482	c
-10.17471629742583	-4.58663651800763	-1.81200803827033	c
-8.73560178221728	-4.60669491405605	0.39944033652177	c
-6.61534695439655	-3.05724988451176	0.60214267356869	c
-4.01327836461735	2.57774218412450	-1.82149347545029	c

-5.68987753076466	4.10376694123476	-0.43282504155683	c
-6.08863961721813	6.64304597705069	-1.01009462117795	c
-4.81447043888930	7.73267934500018	-3.04974746127862	c
-3.17768135318769	6.25917371451082	-4.50317744245129	c
-2.83916947387536	3.71222890455267	-3.91361790512121	c
-1.11723793789296	-0.29556721386028	4.93954190211815	c
-2.34562814435277	2.05325201378988	5.03744878544421	c
-4.10477716560792	2.64028365705481	6.91265935380543	c
-4.63061185802347	0.88753279681884	8.81154586072228	c
-3.38896715200552	-1.44223566409790	8.81466128353930	c
-1.66871754994554	-2.00744269780797	6.90093029085219	c
3.05199909552865	-1.45230769888551	3.82206820330020	c
4.31719038928890	-3.72605318194445	3.26017694052668	c
6.82816513073765	-4.14405163705753	3.95603093600899	c
8.14786288433488	-2.30986091306390	5.31751376079143	c
6.93190737084361	-0.05767737463814	5.96299058316631	c
4.43987682020200	0.34842998628265	5.21380746642419	c

$Al(N(C_6H_2F_3)_2)_3$, C_1

-5.29206295796552	-0.65820321914183	-0.54852678814659	c
-5.00119249004266	-2.35933234830767	-2.55994984949647	c
-6.81728455022539	-4.21523586062756	-2.96584558906338	c
-9.00423284707490	-4.35106926539792	-1.49583225713986	c
-9.31314973886204	-2.57329230513172	0.44081708625076	c
-7.49282323996044	-0.75345070234674	0.93930060809088	c
-3.34567779243723	-2.31756815801638	-3.78546694335760	h
-6.49557231517613	-5.90991060493557	-4.84352934667174	f
-10.76201306385050	-6.12987031492094	-1.93114665581889	f
-11.41533820965835	-2.69902688392178	1.87418189098361	f
-7.76589001285946	0.52998315798733	2.52423540403919	h
0.00008726750997	0.00663523023658	0.02422635201711	al
-3.30203598796798	1.03639209171901	0.08163618106186	n
3.17421536774107	-4.25904098894617	-0.71046521202625	c
2.06776841980208	4.89393861160799	-0.80305273534852	c
-3.97252470936402	3.55413486533665	0.74253048466643	c
5.07305383098563	1.68604724764555	0.54900416696433	c
-1.05358402264320	-5.19833657497052	0.83708603101012	c
4.39138697786838	-3.16455734020724	-2.79518909920002	c
0.39765678445865	5.42060213663957	-2.79275002441256	c

-2.70292124471207	4.79609453489731	2.71033309204921	c
5.55512539684293	0.05477049598178	2.58184559576048	c
-2.64820277897905	-4.69817875723138	2.89469458201820	c
6.87559645627045	-3.82427133115755	-3.34435562974790	c
-0.29655919236563	7.90777618383136	-3.28303435391634	c
-3.14973257261477	7.35087660753499	3.13627384439146	c
7.99576724494194	-0.83376613005180	2.98465585138176	c
-4.61288610206465	-6.35388455617677	3.44966112878452	c
8.15771395173104	-5.65915382731411	-1.94700044919952	c
0.73252638213196	9.92224854153926	-1.92412433605125	c
-4.93605947749968	8.69359577310279	1.73258219333278	c
10.01961199503747	-0.03723156102554	1.49047453503960	c
-4.96563226878850	-8.58530505820845	2.08568869913939	c
6.87826707073192	-6.80790290049670	0.06552288385783	c
2.47569488231359	9.36916050333699	-0.01060996185494	c
-6.26080494180346	7.39801815171243	-0.15728603369524	c
9.52141750067441	1.67419824689403	-0.46609268040889	c
-3.29422477605100	-9.10476423800038	0.10007508289532	c
4.42969779802891	-6.12460053327725	0.70731330087163	c
3.13823052120160	6.90046230857277	0.57334398243194	c
-5.79350188592240	4.87109294797750	-0.67735190996624	c
7.09191870065867	2.52561121667753	-0.96271458558556	c
-1.36967447289172	-7.44595239199935	-0.54692435708202	c
3.46709416972661	-1.74693643273998	-3.97023542107532	h
-0.43735949367962	3.92242548872452	-3.93391749500874	h
-1.31125687122321	3.83108492981715	3.88342489534746	h
4.05041475500957	-0.60254849181502	3.82602758159360	h
-2.43860436479260	-3.00004854130381	4.04244095799497	h
8.07982958591225	-2.70470778953079	-5.29293144813237	f
-1.97432347886076	8.41083714980842	-5.13581887237387	f
-1.86433409012268	8.57139403005229	4.96889328150032	f
8.44633928362801	-2.47680210789376	4.88174156129669	f
-6.20440504346922	-5.82850264919935	5.37124161516360	f
10.54404124628380	-6.30741779517782	-2.52052246115555	f
0.07479407616374	12.31874778127825	-2.44165177394391	f
-5.37576364192402	11.15197122607406	2.18756160271893	f
12.37425443403706	-0.88244934048983	1.92100308994976	f
-6.84699147170399	-10.18803886484405	2.66539489809307	f
8.10602987922766	-8.57605698456248	1.42763603361832	f

3.47141920187090	11.30298379816784	1.31548928991814	f
-7.98484357163795	8.68208732467447	-1.52413804037682	f
11.46425787218063	2.44759808061190	-1.92089237433735	f
-3.62693399559438	-11.25060195207487	-1.23212147111865	f
3.54255281262430	-6.99732888955450	2.34575849141478	h
4.42741187472805	6.55243530306214	2.13874263635651	h
-6.79814474315788	3.96487313613823	-2.22747534734546	h
6.77730760431110	3.77871070360254	-2.56399481182065	h
-0.17520764736003	-7.88031243305589	-2.16482839857035	h
0.75609355782928	-3.36995631925744	0.05743977596401	n
2.54417316284356	2.34882663806816	-0.07850197451969	n

$FAl(N(C_6H_2F_3)_2)_3^-$, C_1

-4.31339339121768	-2.77812958165447	-0.39811907807515	c
-3.06766508069133	-3.83850597657798	-2.49045996719665	c
-3.71152052958770	-6.24714846722011	-3.31512212810783	c
-5.66699188648228	-7.61651327710470	-2.19502946634545	c
-6.96195253227280	-6.50043073396252	-0.17702668848312	c
-6.31770902018260	-4.12880782990060	0.72541159265893	c
-1.50537713367783	-2.86299301428734	-3.40120422462186	h
-2.45658020762103	-7.30095026158213	-5.28294402467611	f
-6.29649276864067	-9.94395636880275	-3.03366933664563	f
-8.85652827565294	-7.82687504132968	0.92939101259237	f
-7.30740792954067	-3.36639694955142	2.36069999870219	h
-0.13405216075929	-0.14446643289481	1.87527431523753	al
-3.47670969018501	-0.47066213765009	0.65749933487163	n
4.08305297966650	-1.92192722761117	-0.99064494646219	c
-0.02842602543760	4.90754109075973	-0.31767490396295	c
-5.26428447132816	1.42571663335983	1.11584263332312	c
3.82405157710159	3.47557643646006	1.67068568035874	c
1.54157438527173	-5.24009499454995	0.84851295266303	c
3.74335533486390	-0.50084512234313	-3.20653930826414	c
-1.59474082108629	4.30522117631162	-2.38128489744163	c
-4.82064052401613	3.23745340848639	3.02563140519917	c
4.84385172719318	2.36725972354291	3.87412268131455	c
-0.11424421799144	-6.04527782197806	2.78710226122706	c
5.83391872560714	0.40865958884462	-4.51180841487052	c
-3.13303410257660	6.14589552384547	-3.44551053557692	c
-6.38521896577782	5.32910178919836	3.22977666567527	c

7.40172929748380	2.61901399807080	4.39568931246255	c
-0.82064782396557	-8.56170741596063	2.93732145182247	c
8.29193951765537	-0.11387562722675	-3.71522131677843	c
-3.08415214612860	8.64742931200082	-2.61398812135967	c
-8.49374468959630	5.65892503392713	1.67547661692651	c
9.02348061983177	3.99756962972093	2.83724774663470	c
0.13161684430460	-10.39405921230402	1.28861721353148	c
8.60715566473008	-1.56790325876952	-1.52885886848598	c
-1.44765330639622	9.24964422121734	-0.62578735391003	c
-8.97398668779380	3.80106941938449	-0.13865346006013	c
7.98152482944217	5.12967116889718	0.68899894616164	c
1.83269807488159	-9.59245323455113	-0.56143218787869	c
6.55442366402881	-2.47096253422292	-0.17419440339174	c
0.05731265729765	7.43982584852158	0.51916248515939	c
-7.41394403888949	1.71919366038917	-0.44197470298442	c
5.44605349121569	4.88675938191534	0.08544662682762	c
2.54162333428083	-7.08098674314243	-0.80968995292137	c
1.86478348766761	-0.01983934227080	-3.88834072055548	h
-1.75191008806085	2.37461739229190	-3.06894901749151	h
-3.23861717822087	3.05184648630882	4.33348183610498	h
3.65475443732830	1.30982675530332	5.18335974993844	h
-0.88093918028712	-4.71644824377830	4.16341405663692	h
5.50755498186008	1.83792163033043	-6.61416441965524	f
-4.70065382412974	5.53164321538844	-5.37715055397714	f
-5.91323788654081	7.09811553934074	5.02491465797155	f
8.38044577189268	1.53377257478340	6.50516450794349	f
-2.45578484083711	-9.31335928024577	4.76554858818194	f
10.30874701880628	0.77911952221564	-4.99712717242515	f
-4.56136238402820	10.43079870070328	-3.69311233941949	f
-10.02883307417255	7.68964077332277	1.93478008595753	f
11.50693977121992	4.25079423489900	3.39445447262216	f
-0.55104049051545	-12.85285227813460	1.49667336331011	f
10.99232299091385	-2.03918606416571	-0.72028419433997	f
-1.39658714488868	11.67481039583255	0.20917539350079	f
-11.01385188268767	4.09724894076583	-1.66803666427777	f
9.53996772996122	6.45172622470443	-0.86306286301382	f
2.77853413418709	-11.34251144644389	-2.18471304040794	f
6.86756316877405	-3.52330038861187	1.56585486526669	h
1.22796868310452	7.97814447502935	2.12350149574674	h

-7.81344915990520	0.39258353943945	-1.96287433695756	h
4.75528043740274	5.72076661483397	-1.66375693541158	h
3.78798215501446	-6.55689691716750	-2.35975667164644	h
1.97664743509236	-2.66107229458054	0.48821973380538	n
1.30319230465282	3.00491490851351	0.98199545135767	n
-0.23868167096456	-0.41842344828298	5.11971802638546	f

$Ga(N(C_6F_5)_2)_3$, D_3

-4.91585012313605	-2.23090050545877	-0.47932539750919	c
-4.34751115105735	-3.77717667868902	-2.55973343662083	c
-5.56763227000642	-6.07599538745367	-2.96558718994691	c
-7.49202606047071	-6.84211117785723	-1.32466745691271	c
-8.14394884973787	-5.31489950550111	0.73191383200309	c
-6.84265644256761	-3.05875376021457	1.15475782425205	c
-2.50861425620336	-3.05794094187152	-4.17936783951479	f
-4.91581737127722	-7.54288274928041	-4.92998437660793	f
-8.68768734186450	-9.04121351891509	-1.70957384474594	f
-9.96699316134037	-6.05681788638206	2.32801295323332	f
-7.42933688753258	-1.67591848638674	3.20725170571069	f
0.00000000000000	0.00000000000000	0.00000000000000	ga
-3.53619160708375	0.00000000000000	0.00000000000000	n
4.38994157261085	-3.14180083510331	-0.47932539750919	c
0.52590855052520	5.37270134056206	-0.47932539750919	c
-4.91585012313605	2.23090050545877	0.47932539750919	c
4.38994157261085	3.14180083510331	0.47932539750919	c
0.52590855052520	-5.37270134056206	0.47932539750919	c
5.44488653385550	-1.87646676070729	-2.55973343662083	c
-1.09737538279815	5.65364343939631	-2.55973343662083	c
-4.34751115105735	3.77717667868902	2.55973343662083	c
5.44488653385550	1.87646676070729	2.55973343662083	c
-1.09737538279815	-5.65364343939631	2.55973343662083	c
8.04578249381516	-1.78371329102875	-2.96558718994691	c
-2.47815022380873	7.85970867848242	-2.96558718994691	c
-5.56763227000642	6.07599538745367	2.96558718994691	c
8.04578249381516	1.78371329102875	2.96558718994691	c
-2.47815022380873	-7.85970867848242	2.96558718994691	c
9.67145512577719	-3.06722930525407	-1.32466745691271	c
-2.17942906530647	9.90934048311130	-1.32466745691271	c
-7.49202606047071	6.84211117785723	1.32466745691271	c

9.67145512577719	3.06722930525407	1.32466745691271	c
-2.17942906530647	-9.90934048311130	1.32466745691271	c
8.67481241519425	-4.39541683824350	0.73191383200309	c
-0.53086356545638	9.71031634374461	0.73191383200309	c
-8.14394884973787	5.31489950550111	-0.73191383200309	c
8.67481241519425	4.39541683824350	-0.73191383200309	c
-0.53086356545638	-9.71031634374461	-0.73191383200309	c
6.07028668155080	-4.39653742852551	1.15475782425205	c
0.77236976101682	7.45529118874009	1.15475782425205	c
-6.84265644256761	3.05875376021457	-1.15475782425205	c
6.07028668155080	4.39653742852551	-1.15475782425205	c
0.77236976101682	-7.45529118874009	-1.15475782425205	c
3.90256166703492	-0.64355320323216	-4.17936783951479	f
-1.39394741083156	3.70149414510367	-4.17936783951479	f
-2.50861425620336	3.05794094187152	4.17936783951479	f
3.90256166703492	0.64355320323216	4.17936783951479	f
-1.39394741083156	-3.70149414510367	4.17936783951479	f
8.99023676428286	-0.48578134925070	-4.92998437660793	f
-4.07441939300564	8.02866409853112	-4.92998437660793	f
-4.91581737127722	7.54288274928041	4.92998437660793	f
8.99023676428286	0.48578134925070	4.92998437660793	f
-4.07441939300564	-8.02866409853112	4.92998437660793	f
12.17376425935202	-3.00315117873362	-1.70957384474594	f
-3.48607691748750	12.04436469764870	-1.70957384474594	f
-8.68768734186450	9.04121351891509	1.70957384474594	f
12.17376425935202	3.00315117873362	1.70957384474594	f
-3.48607691748750	-12.04436469764870	1.70957384474594	f
10.22885473637301	-5.60326033387551	2.32801295323332	f
-0.26186157503264	11.66007822025756	2.32801295323332	f
-9.96699316134037	6.05681788638206	-2.32801295323332	f
10.22885473637301	5.60326033387551	-2.32801295323332	f
-0.26186157503264	-11.66007822025756	-2.32801295323332	f
5.16605642764918	-5.59603523468266	3.20725170571069	f
2.26328045988340	7.27195372106941	3.20725170571069	f
-7.42933688753258	1.67591848638674	-3.20725170571069	f
5.16605642764918	5.59603523468266	-3.20725170571069	f
2.26328045988340	-7.27195372106941	-3.20725170571069	f
1.76809580354186	-3.06243176438385	0.00000000000000	n
1.76809580354186	3.06243176438385	0.00000000000000	n

$FGa(N(C_6F_5)_2)_3^-$, C_3

-4.69358661349006	-1.85645601191411	-0.17878885525076	c
-3.98864340787123	-3.21269585337742	-2.35292715890954	c
-5.13637061208747	-5.49866047076651	-2.99961559553227	c
-7.08117664157931	-6.46725071893227	-1.50530228793759	c
-7.86998319312562	-5.13637348074538	0.63462725160692	c
-6.67709105375402	-2.87524033511688	1.27583481310202	c
-2.18240701458799	-2.30763077270493	-3.88219481116864	f
-4.38891822655716	-6.77032764190268	-5.07784946046105	f
-8.18341690370828	-8.68057567170946	-2.11344502785843	f
-9.73916857762346	-6.07506893377243	2.08973693536507	f
-7.44048679091229	-1.65678770039926	3.37523817110678	f
0.00000000000000	0.00000000000000	1.88407815164387	ga
-3.45911291555942	0.38790769740897	0.57183467817219	n
3.95453137407099	-3.13653723618794	-0.17878885525076	c
0.73905523941908	4.99299324810201	-0.17878885525076	c
-4.97428692332132	2.54426849479257	0.67820037270421	c
4.69054461219940	3.03572459391269	0.67820037270421	c
0.28374231112190	-5.57999308870528	0.67820037270421	c
4.77659792759337	-1.84791859116511	-2.35292715890954	c
-0.78795451972213	5.06061444454255	-2.35292715890954	c
-4.68075096535637	4.42085963714937	2.55327522512652	c
6.16895223501482	1.84321942621248	2.55327522512652	c
-1.48820126965845	-6.26407906336184	2.55327522512652	c
7.33016496051279	-1.69889719793629	-2.99961559553227	c
-2.19379434842535	7.19755766870280	-2.99961559553227	c
-6.09338040081693	6.64368421535741	2.58516723839832	c
8.80028950562968	1.95518011435095	2.58516723839832	c
-2.70690910481277	-8.59886432970836	2.58516723839832	c
9.14139173602820	-2.89885350082650	-1.50530228793759	c
-2.06021509444886	9.36610421975879	-1.50530228793759	c
-7.93876647170914	7.07991846870333	0.75259513392557	c
10.10077248647430	3.33521420486062	0.75259513392557	c
-2.16200601476512	-10.41513267356393	0.75259513392557	c
8.38322151421300	-4.24741863223071	0.63462725160692	c
-0.51323832108733	9.38379211297604	0.63462725160692	c
-8.30816953492540	5.26453041428854	-1.12310527686139	c
8.71330184523238	4.56282066904908	-1.12310527686139	c

-0.40513231030700	-9.82735108333763	-1.12310527686139	c
5.82857669907391	-4.34491030837431	1.27583481310202	c
0.84851435468010	7.22015064349121	1.27583481310202	c
-6.83644299302680	3.07793036862285	-1.16967289841234	c
6.08378738682038	4.38156811917389	-1.16967289841234	c
0.75265560620640	-7.45949848779676	-1.16967289841234	c
3.08967037901121	-0.73620452967811	-3.88219481116864	f
-0.90726336442318	3.04383530238304	-3.88219481116864	f
-2.92837418122459	4.10706237627441	4.37371857939595	f
5.02100744339320	0.48251524458977	4.37371857939595	f
-2.09263326216858	-4.58957762086415	4.37371857939595	f
8.05773484311025	-0.41575085837971	-5.07784946046105	f
-3.66881661655311	7.18607850028236	-5.07784946046105	f
-5.69320912400699	8.37432726292909	4.41349487184092	f
10.09898471130470	0.74330009898288	4.41349487184092	f
-4.40577558729772	-9.11762736191197	4.41349487184092	f
11.60930750302769	-2.74675909251565	-2.11344502785843	f
-3.42589059931942	11.42733476422507	-2.11344502785843	f
-9.33136078649403	9.21598076453110	0.79090259030520	f
12.64695385611964	3.47320511071621	0.79090259030520	f
-3.31559306962563	-12.68918587524730	0.79090259030520	f
10.13074831520031	-5.39683293307488	2.08973693536507	f
-0.39157973757678	11.47190186684727	2.08973693536507	f
-10.04818220907154	5.66537121089102	-2.94379119429860	f
9.93044649503637	5.86929544946526	-2.94379119429860	f
0.11773571403515	-11.53466666035626	-2.94379119429860	f
5.15506363267955	-5.61525672725296	3.37523817110678	f
2.28542315823279	7.27204442765225	3.37523817110678	f
-7.18998214543079	1.47771265366439	-3.13411433428195	f
4.87472777028246	5.48785086386740	-3.13411433428195	f
2.31525437514829	-6.96556351753182	-3.13411433428195	f
1.39361853750000	-3.18963350813780	0.57183467817219	n
2.06549437805943	2.80172581072884	0.57183467817219	n
0.00000000000000	0.00000000000000	5.27446496812404	f

References

[1] Koppang, R. *Acta Chem. Scand.* **1971**, *25*, 3067-3071.
[2] Khvorost, A.; Shutov, P. L.; Harms, K.; Lorberth, J.; Sundermeyer, J.; Karlov, S. S.; Zaitseva, G. S. *Z. Anorg. Allg. Chem.* **2004**, *630*, 885-889.
[3] Altomare, A.; Cascarano, G.L.; Giacovazzo, C.; Guagliardi, A. *J. Appl. Cryst.* **1993**, *26*, 343-350.
[4] Altomare, A.; Burla, M. C.; Camalli, M.; Cascarano, G.; Giacovazzo, C.; Guagliardi, A.; Moliterni, A. G. G.; Polidori, G.; Spagna, R. *Appl. Crystallogr.* **1999**, *32*, 115-119.
[5] Burla, M. C.; Caliandro, R.; Camalli, M.; Carrozzini, B.; Cascarano, G. L.; De Caro, L.; Giacovazzo, C.; Polidori, G.; Spagna, R. *J. Appl. Crystallogr.* **2005**, *38*, 381-388.
[6] Sheldrick, G. M. *Acta Crystallogr. Sect. A* **1990**, *46*, 467-473;
[7] Sheldrick, G. *Acta Cryst. A* **2008**, *64*, 112-122.
[8] Blessing, R. H., *Acta Cryst. A* **1995**, *51*, 33-38.
[9] Coppens, P. (Eds.: F. R. Ahmed, S. R. Hall, P. C. Huber), Munksgaard, Copenhagen, **1970**, pp. 225-270.
[10] Bruker, Bruker AXS Inc., Madison, Wisconsin, USA, **2012**.
[11] Brandenburg, K.; Putz, H.: *Diamond - Crystal and Molecular Structure Visualization v3.2i*, Crystal Impact GbR, Bonn, Germany, 2012.
[12] Ahlrichs, R.; Bär, M.; Häser, M.; Horn, H.; Kölmel, C. *Chem. Phys. Lett.* **1989**, *162*, 165-169.
[13] Treutler, O.; Ahlrichs, R. *J. Chem. Phys.* **1995**, *102*, 346-354.
[14] Becke, A. D. *Phys. Rev. A* **1988**, *38*, 3098-3100.
[15] Perdew, J. P. *Phys. Rev. B* **1986**, *33*, 8822-8824.
[16] Perdew, J. P. *Phys. Rev. B* **1986**, *34*, 7406.
[17] Schäfer, A.; Horn, H.; Ahlrichs, R. *J. Chem. Phys.* **1992**, *97*, 2571-2577.
[18] Eichkorn, K.; Treutler, O.; Öhm, H.; Häser, M.; Ahlrichs, R. *Chem. Phys. Lett.* **1995**, *240*, 283-290.
[19] Eichkorn, K.; Treutler, O.; Öhm, H.; Häser, M.; Ahlrichs, R. *Chem. Phys. Lett.* **1995**, *242*, 652-660.
[20] Eichkorn, K.; Weigend, F.; Treutler, O.; Ahlrichs, R. *Theor. Chem. Acc.* **1997**, *97*, 119-124.
[21] Grimme, S.; Antony, J.; Ehrlich, S.; Krieg, H. *J. Chem. Phys* **2010**, 132, 154104.
[22] Deglmann, P.; Furche, F.; Ahlrichs, R. *Chem. Phys. Lett.* **2002**, *362*, 511-518.
[23] Deglmann, P.; Furche, F. *J. Chem. Phys.* **2002**, *117*, 9535-9538.
[24] By chance scaling factors for BP86/def-TZVP frequencies are very close to unity. Therefore we did not scale the contributions to entropy/enthalpy.
[25] Dunning, T. H. *J. Chem. Phys.* **1989**, *90*, 1007-1023.
[26] Dunning, T. H.; Peterson, K. A.; Wilson, A. K. *J. Chem. Phys.* **2001**, *114*, 9244-9253.
[27] DeYonker, N. J.; Peterson, K. A.; Wilson, A. K. *J. Phys. Chem. A* **2007**, *111*, 11383-11393.
[28] Kendall, R; Dunning, T. H.; Harrison, R. J. *J. Chem. Phys.* **1992**, *96*, 6796-6806.
